# Combined use of olfactory mucosal mesenchymal stem cells conditioned medium and neural guide conduits promotes nerve regeneration in an ovine model

**DOI:** 10.3389/fcell.2025.1598736

**Published:** 2025-05-02

**Authors:** Rui Alvites, Bruna Lopes, André Coelho, Ana Catarina Sousa, Patrícia Sousa, Alícia Moreira, Alexandra Rêma, Maria Simões, Carla Mendonça, Luís Atayde, Justina Prada, Isabel Pires, Bruna Silva, Filipa João, Miriam Metafune, Francesca Bertone, Stefania Raimondo, Nuno Alves, Ana Lúcia Luís, Ana Colette Maurício

**Affiliations:** ^1^ Centro de Estudos de Ciência Animal (CECA), Instituto de Ciências, Tecnologias e Agroambiente da Universidade do Porto (ICETA), Porto, Portugal; ^2^ Departamento de Clínicas Veterinárias, Instituto de Ciências Biomédicas de Abel Salazar (ICBAS), Universidade do Porto (UP), Porto, Portugal; ^3^ Associate Laboratory for Animal and Veterinary Science (AL4AnimalS), Lisboa, Portugal; ^4^ Instituto Universitário de Ciências da Saúde (IUCS), Cooperativa de Ensino Superior Politécnico e Universitário, CRL (CESPU), Gandra, Portugal; ^5^ Centro de Ciência Animal e Veterinária (CECAV), Universidade de Trás-os-Montes e Alto Douro (UTAD), Vila Real, Portugal; ^6^ Departamento de Ciências Veterinárias, Universidade de Trás-os-Montes e Alto Douro (UTAD), Vila Real, Portugal; ^7^ Centre for Rapid and Sustainable Product Development (CDRSP), Polytechnic of Leiria, Marinha Grande, Portugal; ^8^ Associate Laboratory for Advanced Production and Intelligent Systems (ARISE), Porto, Portugal; ^9^ CIPER—Biomechanics and Functional Morphology Laboratory, Faculty of Human Kinetics (FMH), University of Lisbon, Lisbon, Portugal; ^10^ Department of Clinical and Biological Sciences, and Cavalieri Ottolenghi Neuroscience Institute, University of Turin, Turin, Italy; ^11^ Department of Mechanical Engineering, School of Technology and Management, Polytechnic of Leiria, Leiria, Portugal

**Keywords:** peripheral nerve injury, peripheral nerve regeneration, common peroneal nerve, animal model, sheep model, cell-based therapies, olfactory mucosa mesenchymal stem cells, conditioned medium

## Abstract

**Introduction:**

Peripheral nerve injuries remain a significant clinical challenge, particularly in severe neurotmesis injuries requiring complex therapeutic interventions to restore functionality. This study aimed to evaluate the pro-regenerative potential of combining neural guide conduits with conditioned medium from olfactory mucosa mesenchymal stem cells, compared to gold-standard surgical techniques.

**Methods:**

The study was conducted using a validated ovine model of common peroneal nerve injury. Recovery was assessed over 24 weeks through functional, kinematic, ultrasonographic, and electrophysiological evaluations, complemented by post-mortem nerve stereology and muscle histomorphometry.

**Results:**

All therapeutic approaches promoted nerve and muscle regeneration, resulting in notable functional and structural improvements. However, irregularities were observed, as neural guide conduits and conditioned medium did not consistently outperform standard techniques. Additionally, recovery often fell short of normal values in the control group.

**Discussion:**

These findings highlight the complexity of peripheral nerve regeneration in challenging surgical scenarios and underscore the translational potential of biomaterials and cell conditioned medium-based therapies. However, the observed irregularities emphasize the need for further research in complex animal models before application in real clinical cases. Such studies are essential to refine therapeutic strategies, address inconsistencies, and establish cell conditioned medium as a viable tool in peripheral nerve regeneration and repair.

## 1 Introduction

Despite all the important advances achieved in recent years, peripheral nerve injury remains a major medical challenge (Supra and Agrawal, 2023). In addition to the severe functional consequences, the limited efficacy of traditionally available therapies translates into suboptimal results and long-term physical and functional limitations, impacting not only individual health but also the economy (Bergmeister et al., 2020; Kigozi et al., 2019). These injuries are classified according to the severity of nerve disruption, resulting in varying degrees of sensory, motor, and autonomic dysfunction (Lavorato et al., 2023). Neurotmesis, the most severe form involving complete transection of axons, myelin sheaths, and connective tissue coverings, results in total functional loss and an inability of the nerve to regenerate without external therapeutic interventions (Lam and Leung, 2024).

In contrast, in less severe cases such as neuropraxia or axonotmesis, intrinsic regenerative capacity may suffice. Wallerian degeneration clears debris and creates a favorable environment for axonal regrowth and reinnervation of target tissues (Alvites R. et al., 2018; Krishnan et al., 2024). Nevertheless, in real clinical scenarios where injuries are often complex and involve surrounding tissues, therapeutic interventions are typically necessary. Factors limiting effective regeneration include chronic denervation, long nerve gaps, excessive inflammation, scarring, misdirection or neuroma formation, and overall delayed intervention, all contributing to functional loss and pain (Kigozi et al., 2019; Scott et al., 2022; Duraku et al., 2024).

Rapid therapeutic intervention is essential in neurotmesis cases. When end-to-end (EtE) suturing is not viable due to tension or gap length, autologous or allogeneic nerve grafts are used. However, these have limitations, including donor site morbidity and risk of rejection (Ismayilzade et al., 2024; Saffari et al., 2024). Xenografts have also been explored with limited success (Hsu et al., 2023). These constraints have led to the development of biomaterials such as neural guide conduits (NGCs). These tubular structures guide axonal growth and protect against inflammation and neuroma formation, promoting vascularization and proper alignment of regenerating axons (Zheng et al., 2023). Though effective, their high cost restricts widespread use in clinical settings.

New therapeutic strategies increasingly involve combining approaches to maximizing regenerative outcomes (Lopes et al., 2022). A common model uses NGCs with intraluminal administration of adjunct therapies to maintain contact with regenerating tissue. Among these, mesenchymal stem cells (MSCs) are widely studied for their proven efficacy in peripheral nerve repair (Sharifi et al., 2024). MSCs contribute to nerve regeneration through differentiation, neuroinflammation reduction, immune modulation, angiogenesis promotion, extracellular matrix restoration, and support for axonal regrowth and remyelination (Lavorato et al., 2021). Beyond their direct effects, MSCs act via paracrine mechanisms-secreting cytokines, growth factors, and extracellular vesicles–then prompting development of both cell-based and cell-free approaches using their secretome, such as conditioned medium (CM) (Alvites R. et al., 2022). Studies show that CM offers similar regenerative benefits to MSCs, with added technical and economic advantages (Alvites R. D. et al., 2022; Contreras et al., 2022).

Different NGCs and MSC sources have enabled exploration of multiple therapeutic combinations. Our group has focused on MSCs derived from the olfactory mucosa (OM-MSCs), a niche related to a sustained olfactory nerve regeneration (Alvites R. D. et al., 2018). Different studies of cellular characterization (Alvites et al., 2020) and application of MSCs *in vivo* in the rat model, in combination with chitosan-based NGCs, have demonstrated the efficacy of this combination in stimulating the rat sciatic nerve regeneration after injury (Alvites et al., 2021). Likewise, the use of the secretome of these cells after conditioning revealed similar performances after administration into the lumen of NGCs, allowing the effective use of this cell-free approach in the rat model (Alvites R. D. et al., 2022). Our group has extensively characterized the CM components of OM-MSCs and identified several biofactors with the potential to directly participate in the nerve regeneration process (Alvites R. D. et al., 2022; Alvites et al., 2021).

Despite progress, most studies remain limited to *in vitro* or rodent models studies (Lopes et al., 2024) which, although standardized, involve small-scale injuries and species-specific regeneration patterns, hindering clinical translation (Gordon, 2015). Complex animal models are essential to bridge this gap (Contreras et al., 2023; Hellman et al., 2021; Fadia et al., 2020). Our group has developed an ovine model of nerve regeneration considering its anatomical and physiological similarity to humans, including comparable nerve size, distribution, regeneration rate, and nerve polyfasciculation (R et al., 2021). To support its use, we developed and validated a sheep common peroneal neurotmesis model, including surgical protocols, functional, kinematic and ultrasound assessments, and baseline values for healthy nerves (R et al., 2021; Silva et al., 2024; Alvites et al., 2023). Reference stereological parameters were also established. This foundation enables testing of advanced therapies in a translationally relevant large-animal model.

The aim of this study was to test the use of Reaxon® NGCs in combination with OM-MSCs CM to promote regeneration of the common peroneal nerve in the sheep after neurotmesis. A standardized surgical approach was used, and different therapies were applied to compare the performance of a gold-standard technique and innovative approaches. Subsequently, functional, kinematic, ultrasound and electrophysiological evaluations were performed *in vivo*. Finally, *post-mortem*, the level of nerve regeneration was assessed stereologically for the injured nerves and the level of muscle reorganization of the effector muscles was determined histomorphometrically.

## 2 Materials and methods

### 2.1 Animals

All procedures involving animals were previously approved by the Organism Responsible for Animal Welfare (ORBEA) of the Abel Salazar Institute for Biomedical Sciences (ICBAS) from the University of Porto (UP) (project 459/2023/ORBEA) and by the Veterinary Authorities of Portugal (DGAV) (project DGAV: 2018-07-11014510), taking place in facilities previously approved by the official authorities (Clinical and Veterinary Research Center of Vairão—CCIVV). All procedures were performed following the assumptions of Directive 2010/63/EU of the European Parliament, its transcription into the Portuguese DL 113/2013, and the OECD Guidance Document on the Recognition, Assessment and Use of Clinical Signs as Humane Endpoints for Experimental Animals Used in Safety Evaluation (2000). Additionally, all measures were taken to avoid or minimize any discomfort or pain in the animals, considering humane endpoints for animal suffering and distress.

Twenty-seven (*Ovis aries*), merino breed, female gender, 5–6 years and 50–60 kg BW were used in this work, considering animals specifically included in this study and reuse of data from previous ones, to follow the assumptions of reduction and reuse of animals for experimental purposes. The animals were purchased from authorized national producers approved by the host institution and with brucellosis-free (B3) or officially free (B4) health status. The animals were subjected to pre-movement tests for infectious diseases. Additionally, all animals were tested and vaccinated against bluetongue disease. After reception, the animals were assessed for their general health status and subjected to a prophylactic protocol of corrective hoof trimming, internal deworming and vaccination against enterotoxaemia. They were also subjected to 15 days of acclimatization before participating in the planned activities. To ensure their gregarious behavior, the animals were kept in groups of 5/6 animals, were fed with hay and concentrate according to their nutritional needs and had permanent access to fresh water.

Before being surgically intervened, and regularly throughout the work, the animals were subjected to a general physical examination as well as neurological evaluations. Only animals evaluated as healthy in these phases were included and maintained in the study, establishing as exclusion criteria the identification of deviations from the normal health status for the species, significant changes in wellbeing or altered neurological examinations. For surgical intervention, animals were pre-anesthetized with xylazine (Rampun®, Bayer, Leverkusen, Germany, 0.1 mg/kg, IM) and butorphanol (Dolorex®, Merck Animal Health USA, NJ, United States, 0.05 mg/kg, IM) and induced with tiletamine and zolazepam (Zoletil®, Virbac, Carros, France, 3 mg/kg, IM). Surgical maintenance was guaranteed with tiletamine and zolazepam (1.5 mg/kg, IV) and anesthetic recovery was achieved with atipamezole hydrochloride (Antisedan®, Zoetis, 0.025 mg/kg IM). During the surgical procedure, animals received fresh gases at an adapted rate and were closely monitored. After surgery, the animals were treated with anti-inflammatory drugs (meloxicam-Meloxivet®, Duprat, Teresina, Brazil, 0.5 mg/kg, IM, q72 h), analgesics (butorphanol, 0.05 mg/kg, IM) and prophylactic antibiotic therapy (ampicillin—Albipen LA®, MSD Animal Health, NJ, United States, 15 mg/kg, q48 h) for 1 week. After the established study period, the animals were sedated with the protocol described above and then euthanized using an overdose of sodium pentobarbital (Eutasil®, Ceva Animal Health Solutions, Libourne, France, 100 mg/kg IV).

During all phases of the work, the animals were monitored by experienced veterinarians and researchers, and in the intervals between activities they were supervised by handlers properly trained to identify changes worthy of note.

### 2.2 Surgery

#### 2.2.1 Surgical preparation

Following pre-anesthetic induction, the animals were placed in the right lateral decubitus position on the surgical table, to expose the left limb to be operated on. The surgical site was then prepared with trichotomy of the proximal region of the hind limb, thorough cleaning and asepsis, and placement of surgical drapes. A local anesthetic block of the common peroneal nerve was performed, with administration of approximately 2–5 mL of lidocaine (Anestesin®, Medinfar, Lisbon, Portugal, 1.7 mg/kg), in the lateral surface of the hind limb, in the region where the nerve runs obliquely, about 2.5 cm below the tibial lateral condyle.

#### 2.2.2 Surgical access

After the nerve anesthetic blockage, surgical access was performed as previously described (R et al., 2021). Briefly, an incision was made starting at the level of the patella and extending distally along the tibia, in a plantar position, ending approximately 2 cm distal to the tibial crest. After the skin incision, the common peroneal nerve is immediately visible through the fascia, with the biceps femoris muscle appearing underneath. Subcutaneous and atraumatic debridement allows individualization of the common peroneal nerve from neighboring tissues. A ventrocranial detachment of the biceps femoris muscle, adapted to the anatomical characteristics of each animal, can be performed to facilitate exposure of the nerve.

#### 2.2.3 Nerve injury and therapeutic application

**FIGURE 1:**
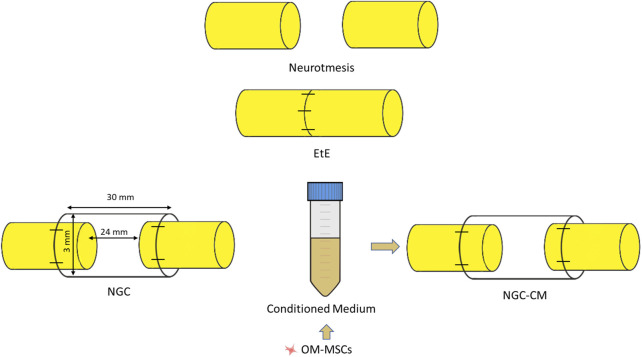
Experimental therapies applied to the common peroneal nerve after neurotmesis injury. The applied NGCs were 30 mm long and had an internal diameter of 3 mm.

After identification and individualization of the common peroneal nerves, the animals were subjected to neurotmesis injuries. The nerves were immobilized using an atraumatic clamp, followed by a complete transection using a scalpel, in a single movement to ensure a clean cut and absence of irregularities and asymmetries in the nerve tops. Following injury induction, three different therapeutic approaches were applied (Figure 1): 1) EtE tension-free suture, in which the nerve tops were aligned and coapted to ensure anatomical alignment and orientation close to the healthy nerve (EtE). The nerve tops were held in position by applying 3 to 4 epineural microsutures with 7/0 monofilament polyglycolic acid material (Safil®), to maintain alignment and avoid rotations (n = 6); 2) application of a 3 cm long and 3 mm diameter NGC (Reaxon® Nerve Guide, Kerimedical, Genève, Switzerland). 3 mm of the nerve tops were introduced into each end of the NGC, leaving a 24 mm gap between them. 3 to 4 epineural sutures of 7/0 monofilament polyglycolic acid material were applied to anchor the nerve tops to the NGC, ensuring alignment and preventing rotation (NGC) (n = 6) or 3) after application of the NGC as described, 15 mL of previously produced and characterized OM-MSCs CM was administered inside the tube lumen, in order to fill it completely (NGC-CM) (n = 4). Animals were selected from the pool of available sheep and randomly allocated to each experimental group using a computer-based random number generator.

The CM used was produced through 48 h cell conditioning as previously described (Alvites R. D. et al., 2022), from cells at P6. These cells had previously been extensively characterized regarding the minimal criteria defined by the International Society for Cellular Therapy, presenting a fibroblastic-like morphology when in culture, being capable of following traditional and neurogenic differentiation pathways and expressing surface markers typical of MSCs (Alvites R. D. et al., 2022; Alvites et al., 2020; Alvites et al., 2021). Likewise, the CM of these cells, used in this work, was widely characterized in previous studies regarding its content of pro- and anti-inflammatory factors, interleukins and growth factors important for the promotion of nerve regeneration (Alvites R. D. et al., 2022; Alvites et al., 2020).

After the induction of lesions and application of the different therapeutic approaches, the nerves or nerve + NGC sets, were carefully accommodated between the muscle masses, with particular care in group 3 to avoid leakage of CM into the surrounding environment. The skin and subcutaneous tissue were sutured with simple interrupted sutures with non-absorbable 4/0 material. Considering the clinical presentation associated with common peroneal nerve injury, a padded bandage was applied to avoid abrasion injuries on the dorsal surface of the foot. Follow-up and recovery times of 24 weeks were considered for the three groups. The peroneal nerves of the contralateral limbs, not intervened, were considered as uninjured controls (UC).

### 2.3 Functional evaluation

After the induction of the lesions and the recovery of the animals, they were functionally evaluated through a neurological examination adapted to the common peroneal nerve and involving the evaluation of motor capacity through observation of movements and posture, proprioception through postural reactions and sensitivity through spinal reflexes. In each of the tests performed, the contralateral right limb was also tested as healthy control. Additionally, the behavioral state of the animals was also determined throughout the study. The components considered during the neurological examination were based on the previously created protocol (R et al., 2021). All animals were evaluated before the induction of the lesion to establish baseline values. After surgery, they were evaluated after 1 week (W1), after 2 weeks (W2) and from then on, every 2 weeks until week 24 (W24).

Posture was assessed with the animals in stationary position, observed in posterior and lateral views to determine the disposition and angulation between the different segments of the digits and the hook. The animals were classified on a scale of 1–5, with 1 corresponding to digits and hock in physiological position with no postural changes, and 5 to severe flexion of digits and extension of the hock, the expected manifestation of a common peroneal nerve injury.

To assess the ability to perform movements, the animals were placed in a large space without obstacles and encouraged to walk and perform movements in a straight line and in circles with variations in direction and speed. The animals were classified on a scale of 1–5, with 1 corresponding to free and agile voluntary movements, with no signs of discomfort and/or pain, and 5 to restriction of movements with signs of discomfort or pain.

Postural reactions were evaluated through proprioceptive assessment, which was determined through static and dynamic proprioceptive positioning tests. In the first case, the dorsal surface of the foot is placed in contact with the ground; in the second case, the foot is placed on a mobile platform that is slowly moved away from the animal, dragging the limb with it and altering the center of balance. In both cases, the time in seconds that the animal takes to reposition the limb to its physiological position is counted. The animals were classified on a scale of 1–5, being 1: <3 s; 2: 3–5 s; 3: 5–10 s; 4: 10–15 s; 5: 15–20 s; 6: >20 s.

Finally, spinal reflexes were assessed using the withdrawal reflex, in which the animal, in lateral position and with the limb to be assessed facing upwards, and using hoof forceps, is stimulated by pinching the skin covering the dorsal surface of the lateral finger and the finger and hoof themselves. The animals were classified on a scale of 1–3, with 1 being the absence of a reflex, 2 the presence of a delayed reflex and 3 the presence of a normal reflex. Reflex identification includes the retraction of the limb after pinching, the manifestation of pain, and the animal’s attempt to look at the stimulus location.

Throughout all tests, the behavioral state was also assessed by determining the animal’s ability to interact with the surrounding environment and the operator and to display typical species behavior.

### 2.4 Kinematic evaluation

To evaluate gait characteristics, the animals were subjected to a kinematic assessment using a three-dimensional reconstruction of the hind limbs, based on gait spatiotemporal variables (namely, flexion/extension, abduction/adduction and internal/external rotation), hip, knee and ankle angles and joint angle coordination measures during the walking cycles. All animals were evaluated before the induction of the lesion to determine the baselines values for kinematic analysis (W0). A new assessment was carried out after the study period (W24).

Kinematic assessment was performed as previously described (Silva et al., 2024). Briefly, to facilitate the application of the markers, the entire left hindquarter of the animal was trichotomized, from the thigh to the feet. An isolated room was prepared to allow the collection of images in a quiet and calm manner. A corridor was placed on the floor to facilitate the orientation of the operator and the animals during the walk. The animals were previously trained to move around the image collection room with an adapted leash, using concentrate food as a stimulus. Twenty reflective markers were placed on the hindlimb at specific anatomical locations to define the bony segment: coxal tuberosity of the iliac wing, ischial tuberosity; greater trochanter of the femur; craniolateral aspect of the femoral diaphysis; femorotibiopatellar joint; caudoproximal aspect of the tibial diaphysis; base of the calcaneus; caudoproximal aspect of the IV metatarsal; metatarsophalangeal joint and lateral aspect of the distal phalanx. Two additional markers were placed at the distal ends of the forelimbs as reference for the contact with the ground. The animals were then encouraged to move around the image capture room, guided by a leash but in a way that did not limit their freedom of movement. The images were collected through 6 infrared cameras (Qualisys Miqus 3, Qualisys AB, Sweden), three placed on each side of the walking corridor, to identify the markers placed on both hindlimbs. The kinematic data was collected using (Qualisys Track Manager), (Qualisys AB, Sweden) operated at a frame rate of 100 Hz. Subsequently a 3D model of the pelvis hindlimbs was built using Visual 3D software (Visual 3D, C-motion Inc., United States). Five body segments were reconstructed using Visual 3D software for biomechanics modeling: pelvis, thigh, leg, foot and metatarsus. Each segment has an embedded three-dimensional coordinate system. The hip joint angle, pelvis orientation, femur orientation, knee angle, and ankle joint angle were determined. Additionally, the spatiotemporal kinematic parameters cycle time, stance time, swing time, step length, step time, double limb support time and gait speed were calculated.

### 2.5 Ultrasound evaluation

To evaluate the characteristics of the common peroneal nerve, of the *tibialis cranialis* muscle and of the surrounding tissues after the induction of the lesion and application of the corresponding therapeutic approaches, an ultrasonographic evaluation was performed. All animals were evaluated before the induction of the lesion to determine the characteristics of the healthy nerve (W0). After surgery, they were evaluated after 4 weeks (W4), after 12 weeks (W12) and after 24 weeks (W24).

Ultrasound scans were performed without the need for sedation, always avoiding aggressive restraint that would increase the animals’ stress. Sheep were placed in recumbency on the surgical table, followed by preparation of the ultrasound field to be evaluated. Whenever justified by the length of the fleece, trichotomy was performed from the gluteal area to the talocrural joint and followed by cleaning with chlorhexidine. The procedure was performed on both the operated and healthy limbs. Acoustic gel was used to improve image acquisition.

Ultrasound scans were performed using the MyLab™ VET ultrasound scanner equipped with an SL1543 linear probe (4–13 MHz, 47 mm) (Esaote®, Genova, Italy). The sciatic nerve was identified as previously described (Alvites et al., 2023). Briefly, the space between the greater trochanter of the femur and the ischial tuberosity was used as a reference point for the site of passage of the sciatic nerve after its emergence from the greater sciatic foramen. The sciatic nerve was followed distally and longitudinally to the site of emission of its two main branches, and the common peroneal nerve was then followed to the most distal limit possible. Nerve diameter was measured after its emission from the sciatic nerve in healthy nerves and in proximity to the lesion site in intervened ones, and each site was measured three times on each animal to minimize the effect of variations in the probe position and in the measurement site. The ultrasound characteristics of each nerve and its relationship with neighboring soft tissues were also evaluated.

Subsequently, the *tibialis cranialis* muscle, as an effector muscle of the common peroneal nerve, was also evaluated ultrasonographically. After its identification as the first muscle mass observed laterally to the tibial crest, in a craniolateral position, the width and thickness were measured in triplicate at mid-muscle belly. The ultrasound characteristics of each muscle and its relationship with neighboring soft tissues were also evaluated.

### 2.6 Electrophysiological evaluation

To determine the level of reinnervation of the cranial tibial muscle as an effector organ of the common peroneal nerve, an electrophysiological evaluation was performed before surgical intervention to establish baseline values (W0), 4 (W4) and 12 (W12) weeks after surgery and at the end of the study period (24W).

To avoid interference in the assessment, electrophysiological evaluation was performed without sedation, always avoiding aggressive restraint that would increase the animals’ stress. Sheep were placed in recumbency on the surgical table, and the procedure was performed on both the operated and healthy limbs. The common peroneal nerve was stimulated using stimulating bar electrodes placed on the skin over the nerve emergence site beneath the *biceps femoris* muscle, using an electromyography (EGM) device (Emg Dantec Keypoint, Medtronic™, Dublin, Ireland). Monopolar recording needle electrodes were placed on the belly of the cranial tibial muscle (active electrode) and on its distal insertion tendon (reference). The ground electrode was placed proximally on the thigh. The compound muscle action potential (CMAP) was assessed by measuring the amplitude and latency at the maximal response observed. To this end, the intensity of the electrical stimulus was progressively increased until a maximum CMAP amplitude was obtained, associated with a contraction of the cranial tibial muscle and movement of the limb.

### 2.7 Nerve stereological analysis

After 24 weeks of functional, kinematic, ultrasound and electrophysiological evaluation, the animals were subjected to a pre-anesthesia protocol as previously described, and then euthanized with an overdose of Sodium Pentobarbital (Eutasil®, Ceva Saúde Animal®, Algés, Portugal; 200 mg/mL, 200 mg/kg b.w., intravenous). Once euthanasia was confirmed, the described surgical approach was used to access and expose the nerves to be harvested. Both the intervened nerves and the healthy contralateral nerves, as controls, were collected. The nerves were fixed and prepared for stereological analysis by light microscopic examination.

After exposure of the nerves, fixation was initiated with the application of a solution consisting of 2.5% purified glutaraldehyde and 0.5% saccharose in 0.1 M Sorensen phosphate buffer at pH 7.4 and maintained at 4°C, to stiffen the nerve and facilitate its manipulation. The nerves were then harvested, including the site of injury/regeneration in the intervened nerves, and kept in the previous solution for a period of 5–10 min, adequately stretched to avoid curling. Finally, the collected segments were immersed in the fixation solution for a maximum of 12 h, after which they were abundantly washed with a solution of 1.5% saccharose in 0.1 M Sorensen phosphate buffer at pH 7.4 and kept immersed in this solution and refrigerated until the time of evaluation. The stereological nerve analysis was performed following the previously described protocol (Alvites R. D. et al., 2022; Alvites et al., 2021; R et al., 2021), and the following parameters were determined: total number of fibers (N), fiber density (N/mm^2^), axon diameter (d,*µm*), fiber diameter (D,*µm*), myelin thickness (M,*µm*) and cross-sectional area (mm^2^). Additionally, the ratios d/D (g-ratio), M/d, D/d were also calculated.

### 2.8 Muscle histomorphometric analysis

Simultaneously with nerve harvesting, the cranial tibial muscles were also collected for subsequent histomorphometric analysis and determination of the level of neurogenic atrophy. After harvesting, the muscles of the operated and healthy limbs were weighed to quantify muscle mass loss. They were then embedded for fixation in 4% buffered formaldehyde and subsequently processed for routine histopathological analysis (hematoxylin and eosin (H&E)). Consecutive 3 μm thick sections originating from the mid-belly region of the muscle were obtained, prepared and stained. Low magnification images (×100) were obtained with a Nikon® (Nikon Corporation®, Tokyo, Japan) microscope connected to a Nikon® digital camera DXM1200 and analyzed with ImageJ® software (Rasband, W.S., ImageJ, U.S. National Institutes of Health, Bethesda, Maryland, United States) through an unbiased sampling procedure. For each individual fiber, the muscle fiber area and the minimum Feret’s diameter (minimum distance of parallel tangents at opposing borders of the muscle fiber) were measured. A minimum of 100 fibers for each study group were analyzed by two experienced operators.

### 2.9 Statistical analysis

Statistical analyses were conducted using GraphPad Prism version 9.00 for Windows (GraphPad Software, La Jolla, CA, United States). Data are expressed as mean ± standard error of the mean (SEM), unless otherwise stated. The sample size (n = 6 per group) was established based on previous studies in nerve regeneration, which demonstrated significant differences with similar group sizes, considering expected effect sizes and variability. This number also reflected ethical and logistical considerations inherent to the use of a large animal model, in accordance with the principles of the 3Rs. Normality of data distribution was assessed using the Shapiro-Wilk test, and data transformation was applied when necessary to satisfy assumptions for parametric testing. Group comparisons were performed using one-way ANOVA followed by Tukey’s *post hoc* test for multiple comparisons. In cases where data did not meet the assumptions for parametric analysis, appropriate nonparametric tests were employed, including for stereological outcomes. Statistical significance was defined as p < 0.05. Levels of significance are indicated as follows: * for 0.01 ≤ p < 0.05, ** for 0.001 ≤ p < 0.01, *** for 0.0001 ≤ p < 0.001, and **** for p < 0.0001.

In the functional tests, ultrasound and electromyography evaluations, the two-way ANOVA test was applied. For kinematic evaluation, a statistical parametric mapping (SPM) was used. SPM was used to analyze the ankle, knee and hip planar angles in gait trials, in a large cohort of sheep after neurotmesis. SPM unpaired t-tests were performed, comparing the mean kinematic angle of experimental group to the respective mean kinematic angle of the control group (α = 0.05). All analyses were performed using open-source SPM1d version M.0.4.7 (2019.11.27; http://www.spm1D.org) in MATLAB.

## 3 Results

### 3.1 Functional evaluation

#### 3.1.1 Behavioral state

No changes in behavioral state were identified in any animal during the evaluation period, and all sheep demonstrated their normal gregarious and environmental exploration behavior, interacting with cohabiting animals and demonstrating awareness of the presence of operators. There was, therefore, no interference with the performance and results obtained in the neurological and functional evaluations.

#### 3.1.2 Postural evaluation–Stationary position

**FIGURE 2:**
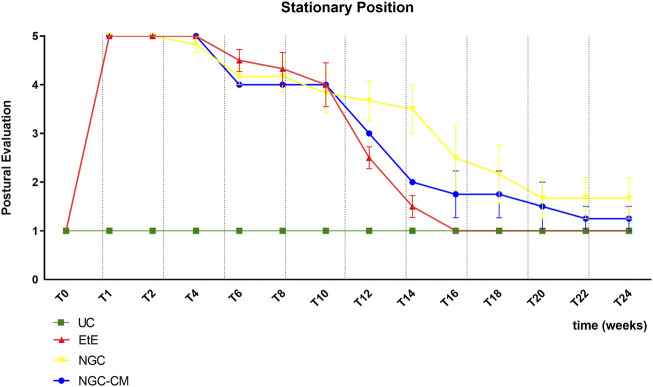
Results of posture evaluation performed in stationary position on all animals over the study period of 24 weeks. UC: Uninjured Control; EtE: end-to-end suture; NGC: application of Reaxon® NGC; NGC-CM: application of Reaxon® NGC and OM-MSCs CM. Classification key - 1: Digits and hock in physiological position, no postural changes; 2: Mild flexion of digits and/or extension of the hock; 3: Moderated flexion of digits and/or extension of the hock; 4: Pronounced flexion of digits and extension of the hock; 5: Severe flexion of digits and extension of the hock. Results presented as Mean +SEM.

The results of the postural evaluation in stationary position can be observed in Figure 2. The complete values of postural evaluation in stationary position can be found in Supplementary Table S1, and the statistical differences observed in T24 in Supplementary Table S2.

**FIGURE 3:**
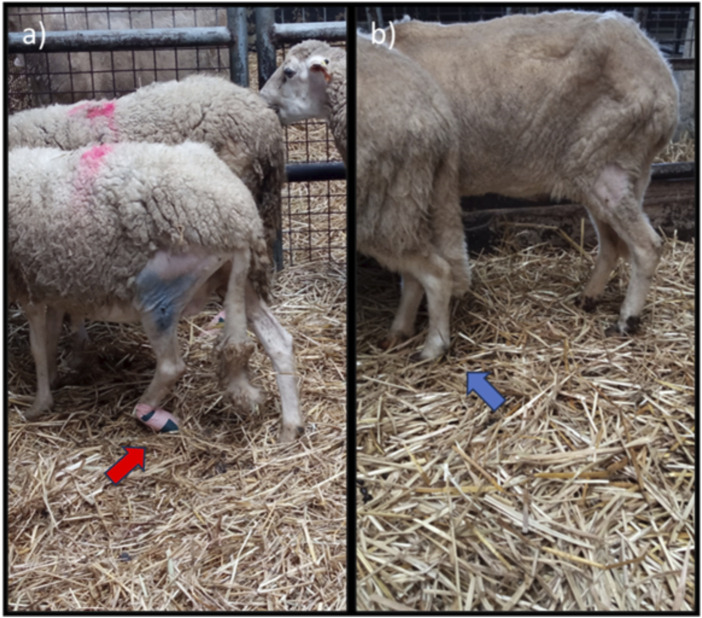
Stationary posture of animals from the NGC group at two different timepoints: **(a)** posture at T1: overflexion of the distal joints and overextension of the hock, with the dorsal surfaces of the digits in contact with the ground (red arrow); **(b)** posture at T24: digits and hock in physiological position, with the plantar surface of the digits in contact with the ground and without postural changes (blue arrow).

Immediately after induction of the neurotmesis injury, regardless of the therapeutic approach selected, all animals displayed the typical posture associated with injury to this nerve, namely, overflexion of the distal joints and overextension of the hock, with the dorsal surfaces of the digits in contact with the ground during the stationary position (Figure 3a). At this timepoint (T), no ability to voluntarily reposition the limb was observed. Progressive improvements began to be observed slowly from T6 after surgery, with a rapid recovery between T10 and T16. Statistical differences were observed between UC and EtE until T12 (*p* = 0.0042) and between UC and NGC (*p* = 0.0152) and NGC-CM (*p* < 0.0001) until T14. At T14 a statistical difference was also observed between EtE and NGC (*p* = 0.0329). The EtE group was therefore the one where a faster recovery of standing posture was observed. In this transitional phase, in addition to the postural recoveries observed, the animals gained the ability to voluntarily reposition their limbs in their physiological position by flicking the foot forward to place the plantar surface in the ground. From T16 onwards, the EtE group presented values close to those observed in healthy animals, with the remaining groups presenting a lower performance, but without statistically significant differences observed between any of the therapeutic groups and the UC one nor between the different therapeutic groups. In the last timepoints the animals presented a posture close to normal, with the digits and hock in physiological position, without postural changes (Figure 3b).

#### 3.1.3 Postural evaluation–In movement

All animals maintained the ability to move freely throughout the monitoring period, performing free, voluntary and stimulated movements, without manifestations of pain or discomfort. At the initial timepoints, due to the direct consequences of the common peroneal nerve injury, during walking/running the animals dragged the limb with the dorsal surface of the digits in contact with the ground, with the bandage applied protecting the extremity and avoiding the risk of skin abrasion. As functional recovery was observed, the animals were able to recover normal posture during walking.

#### 3.1.4 Proprioceptive assessment: static repositioning

**FIGURE 4:**
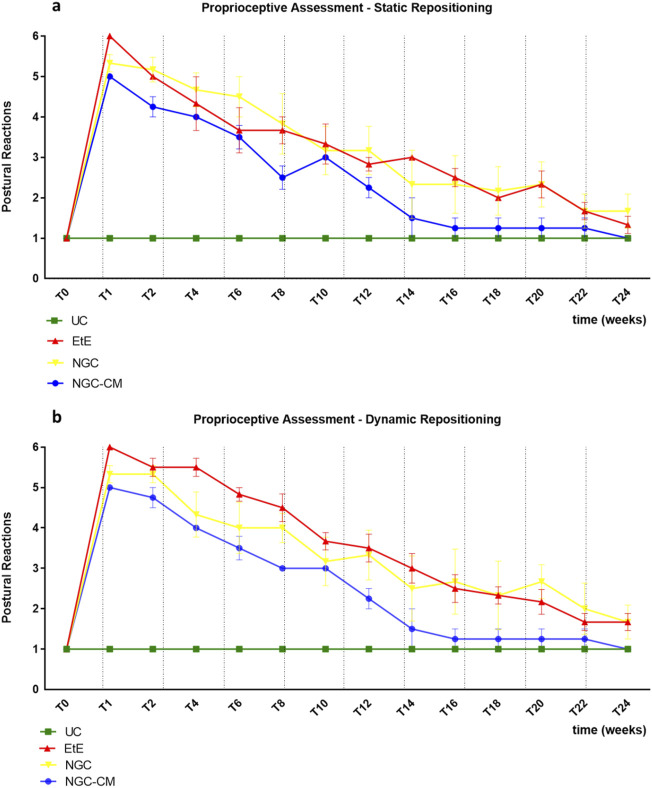
Results of postural reactions evaluation using: **(a)** proprioceptive assessment through static repositioning over the study period of 24 weeks; **(b)** proprioceptive assessment through dynamic repositioning over the study period of 24 weeks. UC: Uninjured Control; EtE: end-to-end suture; NGC: application of Reaxon® NGC; NGC-CM: application of Reaxon® NGC and OM-MSCs CM. Classification key—1: <3 s; 2: 3–5 s; 3: 5–10 s; 4: 10–15 s; 5: 15–20 s; 6: >20 s. Results presented as Mean +SEM.

The results of the postural reactions evaluation using proprioceptive assessment trough static repositioning can be found in Figure 4a. The complete values of postural evaluation in stationary position can be found in Supplementary Table S3, and the statistical differences observed in T24 in Supplementary Table S4.

Immediately after the neurotmesis injury, the functional consequences dictated a total absence of proprioception of the intervened hind limb, with the animals maintaining the limb in the test position for at least 20s without repositioning it (after this period, the test was interrupted, and a total absence of proprioception was considered). From T2 onwards, and consistently throughout the study period, there was a progressive decrease in the time required for the animals to reposition the limb to its physiological position. The UC group no longer showed statistical differences with EtE from T20 (*p* = 0.0370), with NGC from T8 (*p* = 0.0457) and with NGC-CM from T12 (*p* = 0.0455). The EtE group was therefore the one where the slowest proprioceptive recovery was observed, followed by the NGC-CM group and with the NGC group having the fastest recovery. From T22 onwards, statistical differences were no longer observed between the groups. At T24, all groups presented repositioning times very close to the considered normality (<3 s), with no statistical differences between the study groups and the UC group, and with the NGC-CM group presenting the best performance.

#### 3.1.5 Proprioceptive assessment: dynamic repositioning

The results of the postural reactions evaluation using proprioceptive assessment trough dynamic repositioning can be found in Figure 4b. The complete values of postural evaluation in stationary position can be found in Supplementary Table S5, and the statistical differences observed in T24 in Supplementary Table S6.

As in the proprioceptive assessment using a static approach, immediately after the neurotmesis injury, the functional consequences led to a total absence of proprioception of the intervened hind limb, with the animals maintaining the limb in the test position for at least 20 s without repositioning it. From T2 onward, and consistently throughout the study period, there was a progressive decrease in the time required for the animals to replace the limb to its physiological position. The UC group no longer showed statistical differences with EtE (*p* = 0.0450) and NGC (*p* = 0.0387) from T20 and with NGC-CM from T12 (*p* = 0.0455). In this case, the recovery of the NGC-CM group was faster than in the other groups. From T22 onwards, statistical differences were no longer observed between the groups. At T24, all groups presented repositioning times very close to the considered normality (<3 s), with no statistical differences between the study groups and the UC group, and with the NGC-CM group presenting the best performance.

#### 3.1.6 Withdrawal reflex

**FIGURE 5:**
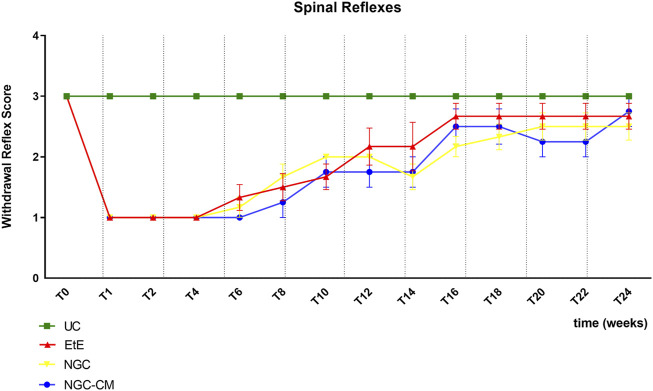
Results of spinal reflexes assessed using the withdrawal reflex over the study period of 24 weeks. UC: Uninjured Control; EtE: end-to-end suture; NGC: application of Reaxon® NGC; NGC-CM: application of Reaxon® NGC and OM-MSCs CM. Classification key - Spinal Reflexes - 1: Absent reflex; 2: Reflex present but delayed; 3: Reflex present. Results presented as Mean +SEM.

The results of the spinal reflexes assessed using the withdrawal reflex can be analysed in Figure 5. The complete values of the spinal reflexes assessed using the withdrawal reflex can be found in Supplementary Table S7, and the statistical differences observed in T24 in Supplementary Table S8.

Immediately after neurotmesis injury, an absence of sensitivity and of withdrawal reflex was observed in all considered groups. From T6 onwards, a recovery of sensitivity was observed in the extremity of the intervened limb with progressive recovery over the study period. The UC group maintained statistical differences with the EtE group until T10 (*p* = 0.0055), with the NGC group until T16 (*p* = 0.0152) and with NGC-CM until T14 (*p* = 0.0455). The EtE group was therefore the one where recovery was fastest, followed by NGC-CM and NGC. From T18 onwards, statistical differences were no longer observed between the groups. At T24, all groups presented sensitivity similar to that of healthy limbs, with a withdrawal reflex present and without response delays. Although at this final stage of evaluation the score observed in the therapeutic groups is lower than that observed in healthy animals, no statistical differences are observed between the groups and the NGC-CM group is the one that presents the best performance.

### 3.2 Kinematic evaluation

**FIGURE 6:**
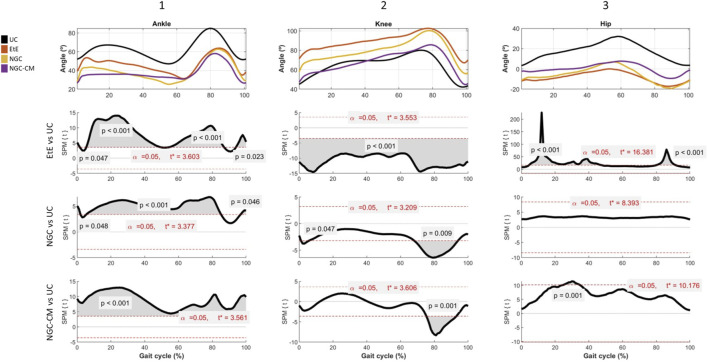
Results of kinematic evaluation performed at 24 weeks. Upper graphs show the ankle (A1), knee (A2) and hip (A3) angles during the stance and swing phases of the gait cycle. The lower graphs show SPM statistic as a function of the gait cycle: (B1), (B2), (B3) - ETS vs. UC for ankle, knee and hip, respectively; (C1), (C2), (C3) – NGC vs. UC for ankle, knee and hip, respectively; and (D1), (D2), (D3) – NGC-CM vs. UC for ankle, knee and hip, respectively. The moments of the gait cycle in which the critical threshold (t ∗) was exceeded are represented by the grey area of the lower graphs.

The results of kinematic evaluation can be analyzed in Figure 6.

Animals in the UC group present the typical movement pattern for a healthy animal. In ankle movements during gait, there is an initial dorsiflexion (0%–10%) during initial contact, a plantarflexion (10%–50%) during the support phase followed by a maximum dorsiflexion (60%–80%) during the foot push-off, and a late dorsiflexion (80%–100%) in the gradual return to prepare the next initial contact. In knee movements, it presents, in sequence, a small flexion (0%–10%) during the initial contact, a gradual extension (10%–50%) during the support phase, a maximum and rapid flexion (50%–80%) during the swing phase and a return to the initial position before the next cycle. Finally, for the hip, an initial extension (0%–50%) is observed during the stance phase and a maximum flexion (50%–100%) during the swing phase to prepare for the next contact. The range of motion is greater compared to that of the knee and ankle joints.

At the ankle level, all experimental groups presented significant changes in the movement pattern, with reduced dorsiflexion, particularly in the swing phase (50%–80% of the cycle) and limited plantarflexion, with the therapeutic groups not reaching the same degree as the UC group. The EtE group presented the most pronounced differences compared to UC, with the curve showing a greater flattening indicative of stiffness or limitation of movements at the ankle. Statistically significant differences (*p* < 0.001) were observed during most of the cycle, especially in the dorsiflexion phases (from 50%–80% of the cycle) and plantarflexion (between 0%–40% of the cycle), and the t values indicate a robust discrepancy, exceeding the critical limit (t∗ = 3.603) in several regions of the cycle. In the NGC group, although the differences regarding UC are also evident, the performance is better. Dorsiflexion is moderately reduced, peak dorsiflexion during the swing phase (50%–80%) is lower than in the control group, but higher than in the EtE group, and plantarflexion is slightly limited. The gait cycle curve presents a fluidity close to normal, but still flatter than in UC. Significant differences (*p* < 0.05) are observed during dorsiflexion (mainly between 50%–80% of the cycle) and plantarflexion (between 20%–40% of the cycle, with lower intensity in relation to EtE). The statistical pattern and t values suggest less stiffness than EtE, but still with relevant changes, with t exceeding the critical limit (t∗ = 3.377) in several regions of the cycle. The NGC-CM group occupies an intermediate position, with evident differences regarding the control group. Dorsiflexion is consistently reduced with a smaller angle throughout the swing phase (50%–80%) compared to UC, and plantarflexion is equally limited and similar to the NGC group. The gait cycle curve is flatter than that observed in UC and NGC, but less severe than in EtE. Statistically significant differences (*p* < 0.05) are observed during dorsiflexion (50%–80% of the cycle) and plantarflexion (20%–40% of the cycle). The statistical pattern is similar to NGC, but with slightly higher t values in some parts of the cycle (t* = 3.561).

At the knee level, the EtE group showed a smaller angular amplitude compared to the UC, especially during the period of greatest flexion (∼40%–70% of the cycle). Maximum flexion was significantly reduced, suggesting a limitation in joint movement or functional adaptation. Statistically significant differences were observed throughout almost the entire gait cycle (*p* < 0.001). The t value consistently exceeded the critical limit (t* = 3.553), indicating a global change in the knee movement pattern. In the NGC group, although smaller amplitudes of movement were also observed compared to the UC, the differences were less severe. Maximum flexion (∼40%–70% of the cycle) was reduced, but the general movement pattern was closer to normal. In this case, statistically significant differences appear at two specific moments of the cycle: at the beginning (∼10%, *p* = 0.047), probably related to the initial stance phase, and at the end (∼80%, *p* = 0.009), probably related to maximum extension before the next cycle. Finally, in the CM-treated group, the curve presents a reduced movement pattern compared to UC, and similar to EtE. Maximum flexion (∼40%–70%) and initial extension (∼0%–20%) are clearly more limited. Statistically significant differences also appear in a large part of the gait cycle, with t consistently exceeding the critical threshold (t* = 3.606) and reinforcing the existence of marked changes in the knee movement pattern.

At the hip level, the EtE group presents a reduced range of motion compared to the UC group, evidencing that maximum extension (∼0%–20% of the cycle) and maximum flexion (∼40%–70%) are significantly limited due to a global restriction in the hip range of motion, which can affect both the stance and swing phases. Statistically significant differences are observed throughout the gait cycle (*p* < 0.001), with the t value exceeding the critical limit (t* = 16.381). In the NGC group, the movement curve is similar to that of the UC group, with subtle differences, namely, in maximum extension (∼0%–20%) and maximum flexion (∼40%–70%), which are slightly reduced compared to the control. In this case, no statistical differences are observed throughout the gait cycle, and t does not exceed the critical limit (t* = 8.393) at any point on the graph. Finally, in the NGC-CM group, notable differences are again observed compared to UC, with a marked reduction in initial extension (∼0%–20%) and maximum flexion (∼40%–70%). The movement pattern is more limited than that of NGC, but less severe than EtE. Significant differences are observed throughout much of the gait cycle (*p* = 0.001), with t exceeding the critical threshold (t* = 10.176) in much of the cycle.

Overall, the kinematic patterns indicate that the ETE and NGC-CM groups present more significant and broader changes in hip and knee movement patterns throughout the gait cycle, reflecting important functional impairments. In contrast, the NGC group demonstrates minimal or statistically non-significant differences, suggesting a greater preservation of normal biomechanics. The application of NGCs led to better functional performance than the standard EtE suture.

The spatiotemporal parameters resulting from the kinematic evaluation at T24 can be found in Supplementary Table S9.

The EtE group presents a gait speed of 0.846 m/s, below that expected for a healthy adult sheep (Silva et al., 2024). The step time on the left side (0.408 ± 0.049s) is slightly higher than on the right side (0.366 ± 0.054s). The left side presents higher values of stance time (0.485 ± 0.078s vs. 0.466 ± 0.106s) and swing time (0.285 ± 0.036s vs. 0.248 ± 0.039s), but the number of steps per minute is higher on the right side (167.430 ± 26.013) than on the left (149.369 ± 20.364). Finally, the double limb support time is 0.229 ± 0.082s.

In the NGC group, gait speed is once again low for the species standard (0.805 m/s), being lower than in the EtE group. Step time on the left side is significantly higher (0.488 ± 0.055s) than on the right side (0.407 ± 0.068s), with the right side presenting longer stance time (0.614 ± 0.092s vs. 0.549 ± 0.110s). The left side presents higher values of sway time (0.333 ± 0.066s vs. 0.294 ± 0.034s) but a lower value of steps per minute (124.473 ± 13.102 vs. 151.638 ± 27.426). Finally, the average double support time is high (0.276 ± 0.156s), with values higher than the EtE group.

In the NGC-CM group, the mean gait speed was lower than normal for the species (0.745 m/s, the lowest value among the three therapeutic groups), and there was also a clear asymmetries between the intervened and healthy sides. The step time was higher in the left side (0.464 ± 0.059s) than in the right side (0.403 ± 0.071s). Stance time was longer on the right side (0.610 ± 0.111 s) than on the left side (0.537 ± 0.084s); swing time was shorter on the right side (0.238 ± 0.036s) than on the left side (0.323 ± 0.064). Likewise, the right side presents a greater number of steps per minute on the right side than on the left (153.605 ± 28.196 steps vs. 131.490 ± 17.221 steps respectively). The double limb support time of 0.304 ± 0.137s can be considered high, although it is the lowest value among the three groups.

The spatiotemporal analysis of the three groups revealed consistent patterns of gait asymmetry resulting from nerve injury, compatible with motor or neurological deficits and possible compensatory biomechanical mechanisms. In general, the right side was consistently used as a compensatory basis, while the left side showed more signs of functional impairment. Considering the spatiotemporal parameters, the EtE group was the one where the final performance of the intervened limb was better at 24 weeks, in opposition to the NGC-CM group with the worst values.

### 3.3 Ultrasound evaluation

**FIGURE 7:**
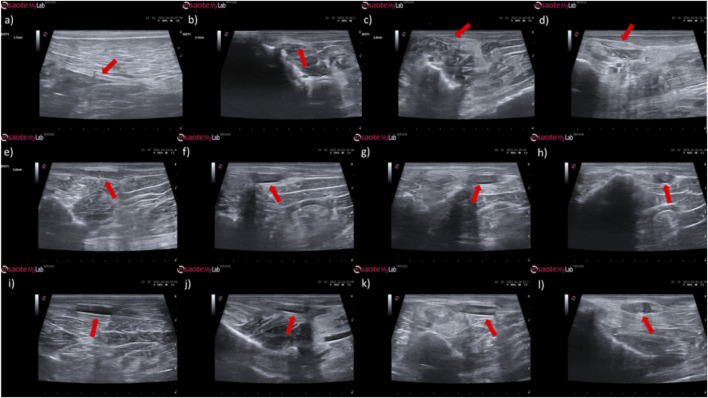
Ultrasonographic appearance of the common peroneal nerves submitted to different treatments. **(a)** intact common peroneal nerve (UC); **(b–d)** nerves that received an EtE suture 1 month, 3 months and 6 months after injury, respectively; **(e–g)** nerves that received a NGC 1 month, 3 months and 6 months respectively; **(h)** nerve that received a NGC 6 months after surgery, transversal section; **(i–k)** nerves that received the combination NGC-CM 1 month, 3 months and 6 months after injury, respectively; **(l)** nerve that received the combination NGC-CM 6 months after surgery, transversal section. In each panel, the red arrow indicates the common peroneal nerve or common peroneal nerve + NGC set.

**FIGURE 8:**
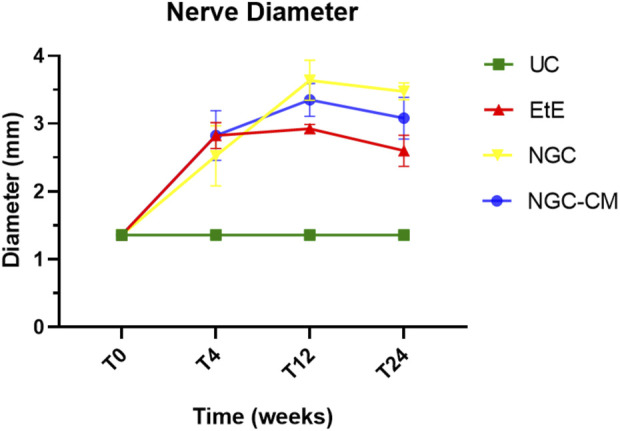
Results of ultrasound measurements of the diameter of the intervened common peroneal nerves over the 24-weeks study period. UC: Uninjured Control; EtE: end-to-end suture; NGC: application of Reaxon® NGC; NGC-CM: application of Reaxon® NGC and OM-MSCs CM. Results presented as Mean ± SEM.

The results of the ultrasound evaluation of the common peroneal nerves can be seen in the Figure 7 and Figure 8 for the ultrasound images obtained and for the results of nerve diameter measurements, respectively. The complete values of nerve diameter measured using ultrasound can be found in Supplementary Table S10, and the statistical differences observed in T24 in Supplementary Table S11.

In animals that received EtE sutures, after 1 month (Figure 7b) an increase in nerve diameter associated with typical swelling due to edema and inflammation observed in the initial phases of the regenerative process was observed. The nerve appears hypoechoic and has a disorganized architecture, where the fascicular pattern of the healthy nerve (Figure 7a) is lost, particularly distal to the lesion and suture site. Hypoechoic areas derived from post-surgical effusion are also observed around the nerve. After 3 months (Figure 7c) the diameter of the nerve increased further, and a hypoechoic appearance with structural disorganization of the nerve continues to be observed, particularly distal to the site of injury and suture. Despite this, perinervous edema appears to have decreased, although some hyperechogenicity associated with the formation of perinervous scar tissue is observed. At 6 months (Figure 7d) the diameter of the nerve remains identical to the previous timepoint, but with evident ultrasound improvements. The hypoechogenicity associated with edema and local inflammation disappears, and a fascicular organization close to normality is observed along the nerve, with the site of injury and suture not being easily distinguished. Some hyperechogenicity associated with scar tissue is still observed, but to a lesser extent compared to the previous timepoint.

In the groups that received NGCs, the tubes are observed as hyperechoic tubular structures, well differented from neighboring tissues, and it is possible to observe the nerve tops introduced inside their lumen. After 1 month (Figures 7e,i) mild hypogeneity was observed around the NGCs (particularly in the NGC-CM group) derived from edema resulting from surgical manipulation. Nerves are observed only at the ends of the NGC, and the lumen of the NGC appears hypoechoic due to the content of inflammatory exudate associated with the normal process of axonal regeneration and regrowth. After 3 months (Figures 7f,j) signs of early continuity are already observed, with a band of nervous tissue occupying the entire length of the NGC, reflecting the continuity of early tissue across the gap. This nervous tissue, however, still appears hypoechoic and without the typical fascicular organization. As the nerves do not occupy the entire internal diameter of the NGC, hypoechoic areas are observed, probably occupied by edematous and inflammatory content. Hypoechogenicity at the periphery of the NGC decreases, being replaced by hyperechogenicity associated with the formation of fibers and scar tissue derived from surgical manipulation and the presence of the NGC. After 6 months (Figures 7g,k) the NGCs are still visible, revealing little or no degradation of the material over the 6 months of implantation. At this timepoint, consolidated nervous continuity is already observed along the entire length of the NGC, with a significant decrease in hypoechogenicity and with the observation of a fascicular organization close to normality. The nerve diameter in the NGC-CM group decreased markedly, not occupying the entire NGC lumen and leaving hypoechoic empty spaces around it. The nerve diameter in the NGC group remains larger. The hyperechogenicity associated with the accumulation of fibrous and cicatricial tissue around the NGCs remains. These features are also visible in a cross section (Figures 7h,l).

When determining the ultrasonographic diameter, an increase in the nerve diameter was observed 1 month and 3 months after the surgical injury and application of therapeutic options. After 6 months, there was a decrease in the diameter of the common peroneal nerves in all groups, more markedly in the EtE and NGC-CM groups, with NGC being the one with the largest final diameter. After 1 month, statistically significant differences were observed only between UC and EtE (*p* = 0.0119), but with the increase in diameter in all groups, after 3 months differences were observed between the control group and all therapeutic groups (*p* < 0.0001 with EtE, *p* = 0.0049 with NGC and *p* = 0.0015 with NGC-CM, respectively). At 6 months, statistical differences remained with the UC group (*p* = 0.0178 with EtE, *p* = 0.0008 with NGC and *p* = 0.0168 with NGC-CM, respectively). Throughout the study period, no statistical differences were observed between the therapeutic groups under study, and at T24 the smallest diameter was observed in the EtE group, followed by NGC-CM and NGC, although in all groups with larger diameters than those of the non-intervened healthy nerves.

**FIGURE 9:**
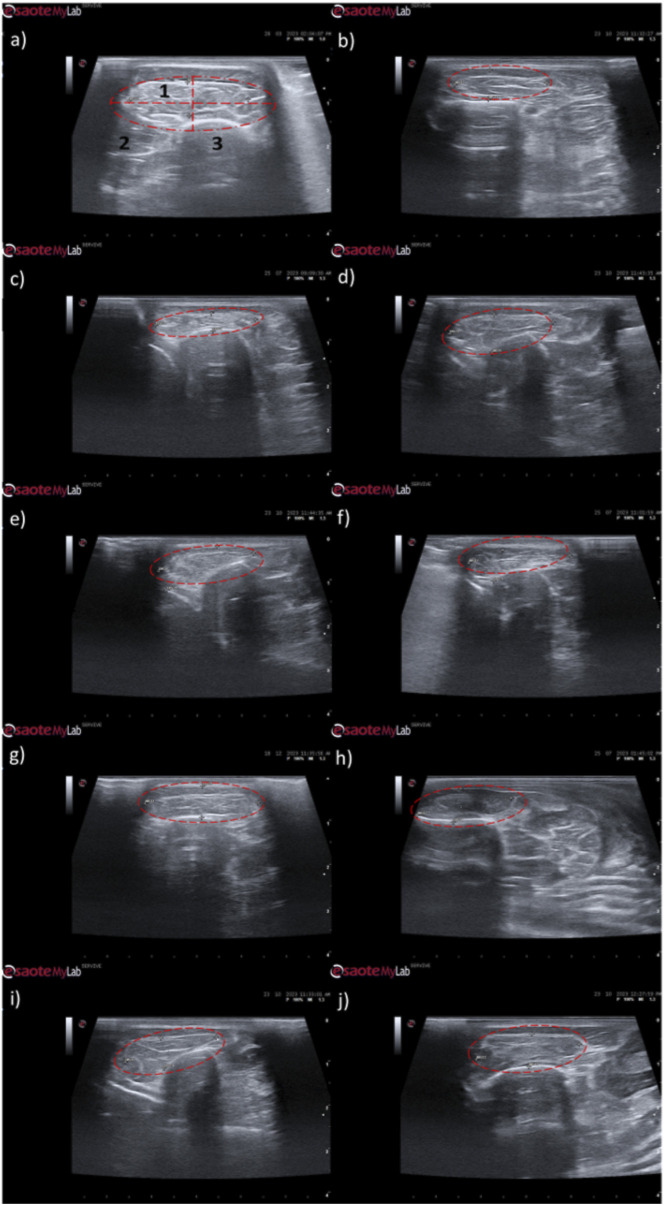
Ultrasonographic appearance of the cranial tibial muscle as an effector muscle of the injured common peroneal nerves subject to different treatments: **(a)** intact common peroneal nerve (UC); **(b–d)** nerves that received an EtE suture 1 month, 3 months and 6 months after injury, respectively; **(e–g)** nerves that received a NGC 1 month, 3 months and 6 months respectively; **(h–j)** nerves that received the combination NGC-CM 1 month, 3 months and 6 months after injury, respectively. The red dashed circle delimits the muscle, in which the thickness (vertical red dashed line) and width (horizontal red dashed line) were measured. 1- Cranial tibial muscle; 2- long digital extensor muscle; 3- tibia.

**FIGURE 10:**
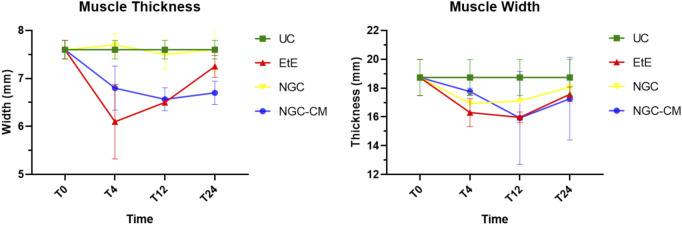
Results of ultrasound measurements of the width and thickness of the cranial tibial muscle over the 24-weeks study period. UC: Uninjured Control; EtE: end-to-end suture; NGC: application of Reaxon® NGC; NGC-CM: application of Reaxon® NGC and OM-MSCs CM. Results presented as Mean +SEM.

The results of the ultrasound evaluation of the cranial tibial muscles can be seen in Figure 9 and Figure 10, for the ultrasound images obtained and for the results of muscle measurements, respectively. The complete values of muscle width and thickness measured using ultrasound can be found in Supplementary Table S12, and the statistical differences observed in T24 in Supplementary Table S13.

Ultrasonographically, significant variations were observed in all study groups over time compared to the UC group, where the muscles presented the expected ultrasound appearance for this muscle, with homogeneous echogenicity and a well-defined fibrillar pattern. After 1 month, due to acute denervation phenomena, hypoechogenicity is observed in all muscles secondary to the expected acute edema and inflammatory infiltration, reflecting the absence of the muscle’s contractile capacity. This ultrasound pattern partially masks the typical fibrillar pattern of the cranial tibial muscles, being particularly evident in the NGC-CM group followed by EtE. At 3 months, the hypoechogenic ultrasound appearance is replaced by a hyperechogenic pattern due to the phenomena of infiltration of fibrous tissue, fat and loss of intracellular fluid. The fibrillar pattern is observed, although in a disorganized way. This phase translates the phenomena of muscle degeneration and repair. The most evident disorganization is observed in the EtE and NGC groups. At 6 months, the muscles present an ultrasound pattern closer to healthy muscle, although still slightly hypoechoic, revealing a reversal of the fibrous, adipose and inflammatory infiltration phenomena. The fibrillar pattern is again easily identifiable, although still disorganized. The NGC and NGC-CM groups present a more organized ultrasound appearance than the EtE group.

One month after the injury, a decrease in muscle width and thickness was observed in all groups. The exception is made in the NGC group, which maintained its thickness stable throughout the 6 months of study. At 3 months, the EtE group had already started to recover its thikness and maintained a stable width. The NGC-CM group presented a lower width and thickness than those measured at 1 month. The NGC group slightly increased width. At 6 months the EtE and NGC-CM groups showed an evident increase in thickness, as well as in width. The NGC group also evidently increased its width. In general, the EtE group had the greatest muscle atrophy 1 month after the injury, but showed good levels of recovery at 6 months (7.60 ± 0.58 mm for thickness and 18.74 ± 3.75 mm for width). The NGC-CM group also had a drop in thickness and width values, with the lowest final values for both parameters at 6 months (6.70 ± 0.42 mm for thickness and 17.27 ± 4.05 mm for width). The NGC group maintained stable thickness values and very close to the control value over the 6 months (7.60 ± 0.65 mm), and in terms of width, although it followed a pattern of evolution identical to the other groups, it also presented the highest value (18.07 ± 0.29 mm) among the therapeutic groups. No statistical differences were observed between the therapeutic groups or between them and the UC group in any parameter or timepoint.

### 3.4 Electrophysiological evaluation

**FIGURE 11:**
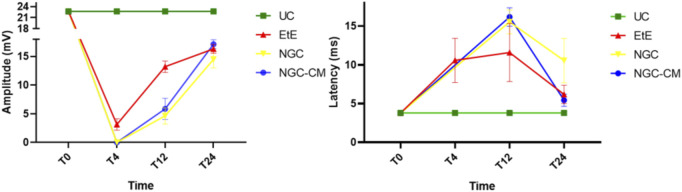
Results of amplitude and latency determined in the cranial tibial muscle over the 24-week study period, through electrophysiological assessment. UC: Uninjured Control; EtE: end-to-end suture; NGC: application of Reaxon® NGC; NGC-CM: application of Reaxon® NGC and OM-MSCs CM. Results presented as Mean +SEM.

The results of latency and amplitude determined through electrophysiological evaluation can be observed in Figure 11. The complete values of latency and amplitude can be found in Supplementary Table S14, and the statistical differences observed in T24 in Supplementary Table S15.

As expected, the amplitude of CMAP decreased in all therapeutic groups 1 month after injury, with the NGC and NGC-CM groups showing no response to the electrical stimulation and the EtE group showing slightly higher values. At this timepoint, statistical differences were observed between the UC group and all other therapeutic groups. At 3 months, an improvement in the amplitude of CMAP contraction was observed in all groups, with EtE maintaining the best results. At this timepoint, statistical differences were not only observed between the UC group and all other groups (*p* = 0.0030 with EtE, *p* = 0.0016 with NGC and *p* = 0.0063 with NGC-CM) but also between EtE and NGC (*p* = 0.0129). At 6 months, the three therapeutic groups showed significant improvement in CMAP amplitude, with the NGC-CM group showing the best performance and the NGC the worst. The values remain, however, significantly lower than those of the UC group (*p* = 0.0027 with EtE, *p* = 0.0290 with NGC and *p* = 0.0066 with NGC-CM).

Regarding latency, 1 month after the injury, an increase in latency time was observed in the EtE group, and no muscular response was detectable in the other groups. At 3 months, with the presence of muscular response in all therapeutic groups, the latency times of the NGC and NGC-CM groups were longer than those of the EtE group, which also increased slightly. At this timepoint, statistical differences were observed between the UC group and the groups that received guide tubes (*p* = 0.0125 with NGC and *p* = 0.0043 with NGC-CM). At 6 months, the latency time of the NGC-CM group suffered an evident decrease, presenting final values close to the EtE group. The NGC group performed worst, but none of the groups showed statistical differences with the UC group.

Overall, the EtE group was the one that showed the best muscular response performance to electrical muscular stimulation after nerve injury and throughout the study period, with muscle response observed even after 1 month. However, after 6 months, the final performance of the NGC-CM group was similar to that of the EtE group. At the last timepoint, the groups did not show differences compared to the UC group in determining latency, but these were observed regarding amplitude.

### 3.5 Nerve stereological analysis

**FIGURE 12:**
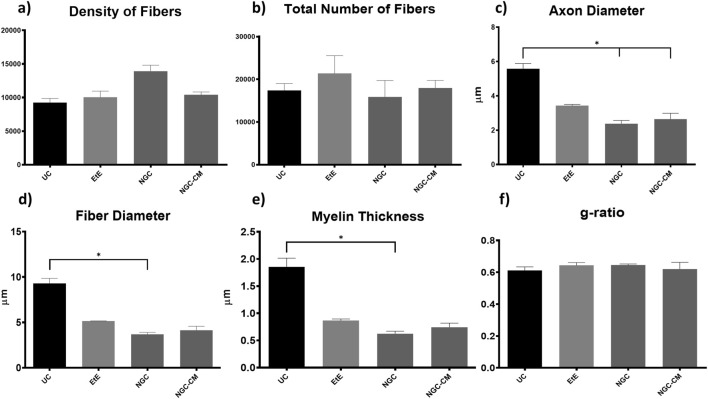
Results of the stereological assessment of the common peroneal nerve 24 weeks after neurotmesis: **(a)** density of fibers; **(b)** total number of fibers; **(c)** axon diameter; **(d)** fiber diameter; **(e)** myelin thickness; **(f)** g-ratio (mean ± SEM)). * corresponds to 0.01 ≤ p < 0.05, ** to 0.001 ≤ p < 0.01, *** to 0.0001 ≤ p < 0.001, and **** to p < 0.0001.

**FIGURE 13:**
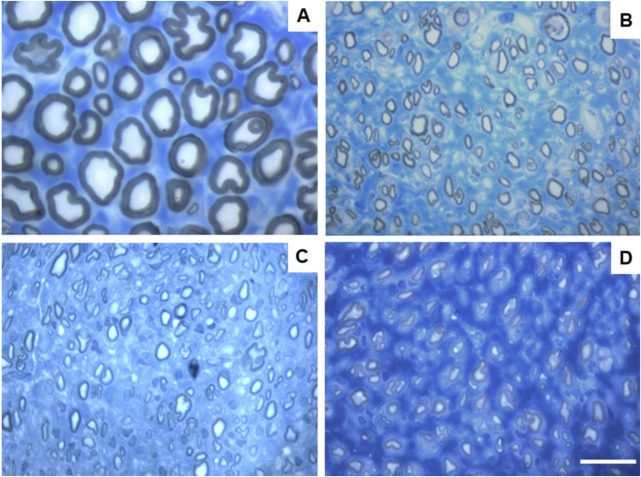
Toluidine blue-stained images of common peroneal nerve semi-thin sections of the different therapeutic groups 24 weeks after surgery: **(A)** UC; **(B)** EtE; **(C)** NGC; **(D)** NGC-CM; Scale bars = 20 µm.

Due to constraints related to the fixation protocol of the collected nerve samples, which will be explored in more detail in the discussion of the article, not all of the collected common peroneal nerves could be analyzed stereologically, which reduced the amount of information that could be obtained in this analysis. The results obtained after the stereological evaluation are shown in Figure 12. The respective stereological images can be found in Figure 13. The total stereological results can be consulted in Supplementary Table S16 and the respective statistical differences in Supplementary Table S17.

After the 24 weeks of study, all the nerves collected and analyzed in the different therapeutic groups show stereological characteristics indicative of nerve fiber regeneration. In direct comparison with the nerves of the UC group, the nerves of the treated groups show microfasciculation phenomena, with axons and fibers of smaller diameter, and a thinner myelin sheath. These results can be observed qualitatively both in the analysis of the different stereological parameters and in the toluidine blue-stained images. Due to the reduced number of viable samples for stereological analysis, despite the evident numerical differences, statistically significant differences were only established in some of the parameters analyzed. Nevertheless no statistical differences can be observed in terms of density and number of fibers between all the groups, some considerations can be made on these parameters. The NGC group presented the highest values of fiber density (13933 ± 1239 fibers/mm^2^), despite also being the group with the lowest number of fibers. The highest number of fiber was observed in the EtE group (21368 ± 7208 fibers). For the parameters axon diameter, fiber diameter and myelin thickness, the results were similar, with the EtE group presenting the best results (3.43 + 0.12 μm, 5.16 ± 0.02 μm and 0.86 + 0.05 μm respectively) and the NGC group the lowest values. In all cases, the values were always lower than those of the UC group. In axonal diameter, differences were observed between the UC group and NGC (*p* = 0.0273) and NGC-CM (*p* = 0.0383). In fiber diameter, differences were observed between the UC group and NGC (*p* = 0.0189). Finally, for the myelin thickness, differences were observed between the UC group and NGC (*p* = 0.0364). The largest final cross-sectional area is observed in the EtE group (2.09 ± 0.39 mm^2^) and the smallest in the NGC one (1.12 ± 0.29 mm^2^), and no statistical differences were observed compared to the UC group (1.94 ± 0.6 mm^2^). Finally, regarding the g-ratio, the EtE and NGC groups had similar results (0.65 ± 0.03 and 0.65 ± 0.00 respectively), only slightly higher than the values of the NGC-CM (0.62 ± 0.05) and UC (0.61 ± 0.05) groups, and with no identifiable statistical differences between the therapeutic groups.

### 3.6 Muscle histomorphometric analysis

**FIGURE 14:**
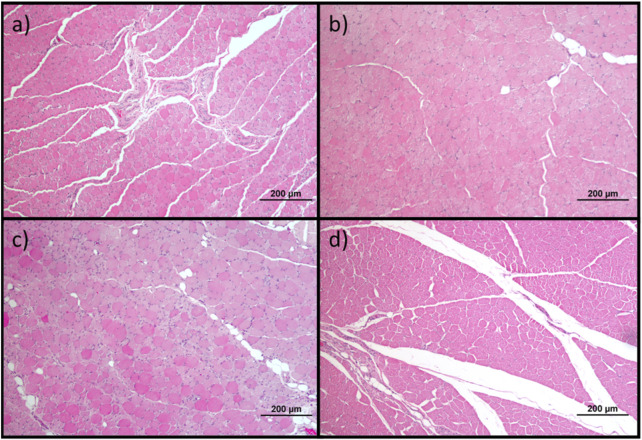
Histological images of the cranial tibial muscles subjected to histomorphometric analysis in the different groups: **(a)** UC; **(b)** EtE; **(c)** NGC; **(d)** NGC-CM; Magnifications: ×100.

**FIGURE 15:**
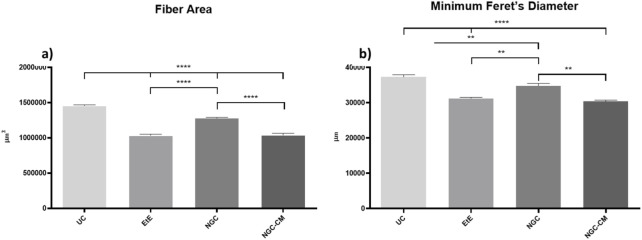
Histomorphometric analysis of cranial tibial muscle: **(a)** individual fiber area; **(b)** minimum Feret’s diameter of the muscle fibers (mean ± SEM). ** to 0.001 ≤ p < 0.01, and **** to p < 0.0001.

The results obtained after the histomorphometric analysis are shown in Figure 14 and Figure 15. The total histomorphometric results can be consulted in Supplementary Table S18 and the respective statistical differences in Supplementary Table S19.

24 weeks after the neurotmesis lesion of the common peroneal nerve, all groups presented muscle fiber area and minimum Feret’s diameter values lower than the control group. Regarding fiber area, among the therapeutic groups, the NGC group was the one that ended with a higher value, followed by NGC-CM and EtE. In this case, statistical differences are always observed between the UC group and the therapeutic groups (*p* < 0.0001) and also between NGC and the remaining groups (*p* < 0.0001 for both cases). The minimum Feret’s diameter results followed a similar organization, with NGC presenting the highest final value again, followed by NGC-CM and EtE. The UC group presents statistically significant differences with EtE and NGC-CM (*p* < 0.0001) and also with NGC, but smaller in this case (*p* = 0.0075). NGC also presents differences with EtE (*p* = 0.0094) and with NGC-CM (*p* = 0.0016).

**FIGURE 16:**
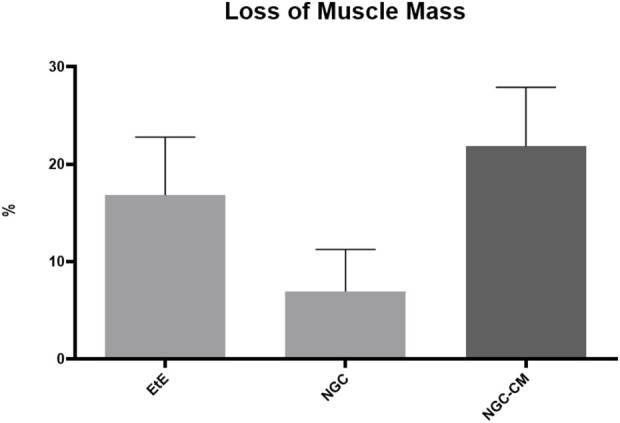
Percentage of muscle mass lost in each therapeutic group as a function of contralateral healthy muscle weight (mean ± SEM).

The percentage of muscle mass lost in the cranial tibial muscles of each therapeutic group, compared to the contralateral control muscle, is shown in Figure 16. The total muscle mass loss results can be consulted in Supplementary Table S20.

The average final weight of cranial tibial muscles harvested for each group was 57.56 ± 4.45 g for the EtE group, 63.37 ± 3.60 g for NGC and 60.51 ± 0.92 g for NGC-CM, as opposed to a value of 77.55 ± 31.90 for the UC. Comparing the final weight of the muscles associated with the injured nerve with those of the healthy limb within the same group, a percentage of muscle mass loss of 16.82% was observed for the EtE group, 6.95% for NGC and 21.84% for NGC-CM. The NGC group, where the highest values of fiber area and minimum Feret’s diameter were observed, was concordantly the group where the final muscle weight after the regeneration period was higher, and as such, where the loss of muscle mass was lower.

## 4 Discussion

In recent years, advances made in regenerative medicine, particularly those applied to the peripheral nervous system, have been remarkable (Fakhr et al., 2024). The new therapies explored and referred to in the literature are multiple, ranging from pharmacological therapies (Bolandghamat and Behnam-Rassouli, 2020), nutritional therapies (Muratori et al., 2022), genetic therapies (Eggers et al., 2020), the use of cellular and cell-based therapies (Contreras et al., 2022; Aisaiti et al., 2024) and the use of innovative biomaterials (Kaplan and Levenberg, 2022). Despite notable advances made in *in vitro* and *in vivo* experimental models in all these areas of applied scientific research, the approaches used in clinical practice and in real medical settings do not appear to have evolved at the same rate. Traditional surgical methods involving EtE sutures or the use of nerve grafts, mainly autologous, remain the most mainstream choice (Nuelle et al., 2022). This difficulty in taking the advances achieved in fundamental and applied research into clinical and surgical practice reveals the complex challenges associated with translating innovative therapies from the laboratory to the surgical table. The use of experimental therapies developed *in vitro* and *in vivo* faces several challenges to be translated into current clinical use. Most of the studies developed *in vivo* are carried out in low-complexity animal models such as rodents, and their exploration in larger and more complex models, to create an intermediate bridge with humans, is rare (Lopes et al., 2024; Alvites et al., 2024). The significant biological differences observed between the most common animal models and humans in parameters such as anatomy, physiology, immune response and nerve regeneration rate will necessarily translate into different responses to established therapies, requiring more studies that often are not performed and frequently lead to dead ends for therapies with high potential.

Over the last decade our group has been exploring new combined therapies using MSCs and their secretion products in conjunction with commercially available biomaterials to confirm the potential of these innovative treatments to promote peripheral nerve regeneration (Lopes et al., 2022). Following a logic of sequential exploration, work has been developed *in vitro* (Alvites et al., 2020) and in the rat model (Alvites et al., 2021). Particularly promising results were achieved when testing the therapeutic combination between OM-MSCs with chitosan-based NGCs (Reaxon®), both using the cells *per se* and using their CM (Alvites R. D. et al., 2022; Alvites et al., 2021). From a translational and scale-up perspective, the group has recently also developed efforts to overcome the reduced availability of works developed *in vivo* in more complex animal models (Contreras et al., 2023), having developed and validated a peroneal nerve injury model in the sheep that includes new protocols for surgical injury induction and for functional, kinematic and ultrasonographic evaluations (R et al., 2021; Silva et al., 2024; Alvites et al., 2023), having established control values for the species both for *in vivo* and *post-mortem* histomorphometric and stereological evaluations. These combined advances made it possible to apply and explore in this work the therapeutic potential of the combination between CM produced from OM-MSCs and chitosan-based NGCs in the ovine model, an alternative that had not previously been studied in this species or in other complex animal models. It is important to note that in this work, CM originating from rat cells was used in a sheep model. This xenogeneic application is possible due to the use of paracrine products of the cells and not by the cells *per se*, which means that the soluble factors, microvesicles, and exosomes present in the CM, as they do not have histocompatibility antigens, are incapable of triggering rejection reactions in the recipient individual, making this cell-free approach safer than the use of cells (Vizoso et al., 2017; Harrell et al., 2019). Furthermore, the functional conservation of paracrine factors released by MSCs, which are highly conserved between mammals and between different species, can promote similar biological effects such as inflammatory modulation, neuroprotection, and axonal regeneration (Műzes and Sipos, 2022; Han et al., 2022) even when used between distinct mammals. Although the secretome was not newly characterized in this study, its composition has been previously described and validated by our group using the same OM-MSC source and conditioning protocol (Alvites R. D. et al., 2022; Alvites et al., 2020). The secretome was shown to contain a rich array of neuroregulatory and immunomodulatory factors, including IL-10, IL-4, IL-6, VEGF, NGF, BDNF, GDNF, HGF, bFGF and TGF-β, among others, which are known to contribute to peripheral nerve regeneration through distinct mechanisms. These factors promote axonal sprouting, Schwann cell activation, revascularization, extracellular matrix remodeling, and inflammation resolution - key processes for functional recovery following neurotmesis. While their individual levels were not remeasured in this experiment, their previously established presence in this standardized CM supports the interpretation that they likely contributed to the observed regenerative outcomes.

Functional assessment and its different components allow quantifying the functional consequences associated with a given nerve injury and monitoring the recovery and therapeutic effectiveness of the instituted treatments over time, providing essential data on the impact of the injury on the peripheral nervous system function and the ability of the nerves to regenerate after treatments. In this work, motor activity was assessed through postural assessment, proprioception was evaluated by determining the repositioning capacity, and nociceptive spinal reflexes were assessed using the withdrawal Reflex. Additionally, the mental state of the animals was also monitored throughout the study period. In all parameters evaluated, functional progression exhibited a consistent pattern of recovery. Immediately after inducing the neurotmesis injury, animals presented the typical symptoms associated with common peroneal nerve injury, including overflexion of the distal joints and overextension of the hock. This resulted in the dorsal surfaces of the digits to be in contact with the ground, both in static positions and during gait, where dragging of the digits was evident. From the fourth week onward, a steady and continuous functional recovery was observed across all parameters and throughout the entire study period. All therapeutic groups followed a similar trajectory of improvement. Notably, the EtE group demonstrated superior results in postural assessment, and the NGC-CM group showed the best recovery in postural reactions and withdrawal reflex. Despite these variations, all therapeutic groups exhibited lower final values compared to the control group in all tests. However, no statistically significant differences were found between the UC and therapeutic groups, nor among the therapeutic groups themselves. By the 24-week mark, all animals demonstrated a near-normal posture, with physiological limb positioning, rapid proprioceptive repositioning, and a robust nociceptive response. Additionally, no significant changes in mental status were observed throughout the study. These findings indicate that the application of NGC or NGC-CM effectively promoted functional recovery comparable to the gold-standard EtE suture, achieving a final performance close to normality. The pro-regenerative effect of the CM may bring slight therapeutic advantages in the functional recovery compared to the use of NGC alone. Functional recoveries following similar patterns in the ovine model have been described previously (Contreras et al., 2023; R et al., 2021), but application of similar therapies in the rat model led to greater differences regarding the gold-standard EtE (Alvites R. D. et al., 2022).

The kinematic assessment of the hindlimb includes the study and description of the movement of the joints and bone segments of this anatomical region, allowing the analysis of the angular variation of different joints during gait and the rhythmic characteristics of the movement (Silva et al., 2024). Thus, it is possible, in a more sensitive way, to understand how nerve injuries translate into specific mechanical changes that often do not resolve with the same effectiveness as the findings identified in the functional assessment, often being associated with adaptive mechanisms rather than an effective recovery. The kinematic assessment made it possible to observe different recovery patterns and outcomes in the therapeutic groups. The main expected consequences of an injury to the common perineal nerve include a loss of the ability to perform dorsiflexion and compensatory changes in the spatiotemporal parameters of gait (Silva et al., 2024; Capodici et al., 2022). At 24 weeks of study, the NGC group was the one that presented a kinematic pattern closest to that of the UC group, presenting a more fluid gait cycle and with fewer statistical differences compared to the control group. The NGC-CM group presented greater limitations, with more marked reductions in joint angles in all phases of gait. The EtE group was the one that presented greater biomechanical restrictions, with persistent stiffness and reduced range of motion across all joints, particularly the ankle and knee, throughout the gait cycle. Regarding the spatiotemporal parameters, however, EtE performed better than the other groups, revealing a walking rhythm closer to normal. Here the NGC-CM group showed the most pronounced asymmetries, reflecting worse recovery and less effective compensatory adaptations. Despite having presented the best joint kinematics results, the NGC group presented an intermediate position in spatiotemporal parameters. The evaluation of these results in parallel with those obtained in the functional evaluation demonstrates the complexity associated with recovery after nerve injury, where the results observed in the different evaluations are not always consistent (Alvites et al., 2021). The NGC group achieved the best outcome in the joint kinematic assessment, again revealing that the application of NGC can promote an effective functional recovery. The addition of CM in this case does not seem to have promoted a more effective recovery in the NGC-CM group. The EtE group, on the other hand, had the best performance in the spatiotemporal parameters although it had the worst in the joint kinematics. These results confirm that different therapeutic strategies can target different aspects of functional recovery, highlighting the advantage of developing and establishing personalized approaches for each type of injury and functional consequence. These types of differences had already been identified in studies previously carried out in the rat model (Alvites et al., 2021). Regardless of the results of each therapeutic group, the performance was always inferior to that observed in the UC group at 24 weeks, with significant statistical differences remaining in several parameters. With a less effective recovery than that observed in the functional evaluation, the kinematic assessment allows to understand in more detail the remaining consequences associated with the nerve injury, reinforcing the importance of the assessment to always be multimodal and never based exclusively on one type of technique. A progressive evaluation over the study period, which was not possible to carry out in this work, would have allowed obtaining more information regarding the variations in kinematic recovery in a sequential manner over the 24 weeks.

The use of ultrasound proved to be an advantageous technique for the non-invasive evaluation of the intervened nerves (Alvites et al., 2023; Lawande et al., 2014) and their respective effector muscles (cranial tibial muscle) throughout the study and the regeneration/reinnervation period, complementing the information obtained about the regenerative process over the 24 weeks. Over the course of the study, notable ultrasound variations were observed in the intervened nerves, particularly in their diameter and fascicular organization. The diameter in all therapeutic groups increased 1 month after surgical intervention, due to the expected phenomena of edema, inflammation and accumulation of exudates resulting from initial Wallerian degeneration. This increase in diameter was slightly higher in the EtE and NGC-CM group compared to the NGC group, in the first case probably due to the absence of a physical barrier provided by the NGC that facilitates a more exuberant inflammatory response, and in the second case due to the presence of CM which may have accelerated the Wallerian phenomena compared to the NGC counterpart. At this stage, an expected hypoechogenic and disorganization of the fascicular pattern of the nerve were also observed and, of course, in the groups that received NGC, there was an absence of nervous continuity. After 3 months, a new increase in nerve diameter was observed in all groups, this time already associated with more advanced stages of Wallerian degeneration, with the formation of Büngner bands on the periphery of the regeneration site. Compared to 30 days, in the EtE group the increase was smaller, since direct contact between the nerve tops limits the need for a longer regenerative process, attributing the increase in diameter to the formation of some perinervous scar tissue. In the NGC-CM groups, and especially in the NGC one, the increase in diameter is much more evident, associated with a more intense regenerative phenomenon necessary for the growth of new axonal sprouts in the gap between the two nerve ends. At this stage, nerve continuity of all nerves was already observed, although some fascicular disorganization and hypoechogenicity persisted, associated with the fluids derived from the regenerative process. Finally, after 6 months, with the entry into the subacute phase of regeneration, there was a reduction in axonal diameter in all groups, even though the final diameter is still greater than that observed in the control groups with significant statistical differences. This is likely associated with the resolution of edema and inflammation as phagocytosis of all cellular and myelin debris occurs. The nerves also present better fascicular organization with reduced hypoechogenicity. Having not suffered such an intense increase in diameter, the EtE group also has the smallest dimensions at 24 weeks. The NGC group is the one where a higher final diameter is observed, with the NGC-CM occupying an intermediate position. Comparing the two groups that received biomaterials, the presence of CM seems to have accelerated the regenerative process, probably through inflammatory modulation phenomena and greater stimulation of the proliferation of Schwann cells, with this group presenting a final diameter closer to normal than the use of NGC alone. In any case, no statistical differences were observed between the three therapeutic groups, which have similar performance. This increase in nerve diameter with subsequent decrease after therapeutic action has already been described previously (Zaidman and Pestronk, 2014). But it is important to understand that in this case reference is made to the diameter of the nerve as a whole, and not to the diameter of the axonal fibers individually, in which some studies indicate that even with the regeneration process completed, there may not be a return to normal pre-injury dimensions (Muratori et al., 2012).

Muscle atrophy after a nerve injury occurs due to the interruption of the connection between the nerve and the muscle, which leads to the loss of nerve stimulation that is essential for maintaining muscle tone and mass. The progression of atrophy depends on several factors, including the severity of the injury, the type of nerve affected and recovery time (Yadav and Dabur, 2024). The phenomenon of muscle atrophy was observed in all therapeutic groups over time. One month after the nerve injury, there was a decrease in thickness and width in all therapeutic groups, except in the NGC group, which maintained its width stable. In this phase, atrophy is directly linked to the acute phase of denervation, where the lack of nerve impulses reduces muscle activity and inhibits trophic factors, causing loss of tone and the beginning of a reduction in muscle volume and contractile capacity. It’s called disuse atrophy. In the ultrasound, this phase is characterized by a typical hypoechogenic pattern of acute edema and inflammatory infiltration, with changes in the typical fibrillar pattern of the muscle. At 3 months, the dimensions of all muscles continue to be reduced, although in some groups there is already recovery of dimensions (EtE and NGC) and in others the reduction continues (NGC-CM). In the first two groups, a gradual reinnervation appears to be already being established, and a reversal of the state of degeneration towards repair, with a return to the contractile capacity of the muscles and dimensional and functional recovery. The ultrasound appearance is also improved, although phenomena of some muscle replacement by fibrous or fatty infiltration are observed, which can delay muscle regeneration (Wang et al., 2024). Despite their smaller dimensions, ultrasonographically the NGC-CM group appears to present better tissue reorganization. Finally, after 24 weeks there is significant dimensional recovery in all groups, with the NGC group with the higher thickness and width and the NGC-CM group with the smallest. In the chronic phase of muscle regeneration, there is a progressive restoration of the dimensions and contractile capacity of the muscle. Ultrasonographically, although some hypoechogenicity is still observed, there is a reversal of the fibrous, adipose and inflammatory infiltration, with a fibrillar pattern that is easier to identify, especially in the groups that received NGC. The final dimensions of the muscles, although smaller than those of the control group, are similar and no statistically significant differences are observed either between UC and the remaining groups, nor between the 3 therapeutic groups. A similar pattern of evolution of muscle dimensions determined by ultrasound was previously described in the ovine model after injury and repair of the common peroneal nerve (Contreras et al., 2023).

Electrophysiological evaluation represents a set of nerve and muscle monitoring through electrical activity, being used in cases of nerve injury to diagnose, locate and monitor the severity of a nerve injury, the functional consequences and its recovery. This technique allows evaluating the electrophysiological properties of nerves undergoing regeneration, including the amplitude and latency of effector muscles, offering a direct measurement of nerve functionality (Shigapova and Mukhamedshina, 2024). In this study, the electrophysiological parameters evaluated, latency and amplitude, reveal different recovery patterns between the therapeutic groups. One month after neurotmesis. no response to electrical stimulation was observed in the groups that received NGC, with an amplitude close to zero in the EtE group. In the case of the NGC and NGC-CM groups, since after 1 month a reconnection between the nerve tops has not yet been established (as observed in the ultrasound evaluation) it is not expected to observe an electrical muscular stimulation and response. In the EtE group, the presence of an earlier detectable CMAP suggests that axonal regeneration was initiated within the first 4 weeks post-injury, allowing a minimal but functional reinnervation of the muscle. While this is consistent with previous studies in peripheral nerve regeneration, it contrasts with the ultrasound findings, which did not indicate complete structural continuity at this stage. This apparent discrepancy may be due to the sensitivity differences between the techniques, as electrophysiology detects functional reconnection at a cellular level, whereas ultrasound may not yet visualize fine regenerating axons within the nerve tissue. At 3 months, improvements in the amplitude of CMAP were already observed in the three groups, reflecting the nerve reconnection established in the NGC and NGC-CM groups, with the EtE group showing higher values as it is in a more advanced phase of nerve regeneration and muscle recovery. After 24 weeks of study, all groups present identical amplitude values, although the NGC-CM group is the one that presents better results, confirming that despite the initial delay associated with the gap left between the nerve tops, the application of biomaterials and combination with CM can promote equally effective muscle reinnervation. In either case, the final amplitude values are still lower than those found in the control group, with statistical differences, demonstrating that none of the therapeutic options can promote a return to normality in this parameter. There are no differences between the therapeutic groups. Latency recovery follows an identical evolution, with no muscular response after 1 month for the groups receiving biomaterials and with the NGC-CM group showing the lowest values after 24 weeks. In this case, no statistical differences were observed with the control group, with the application of the combination of biomaterials and CM surpassing traditional methods in long-term functional outcomes. These results seem to demonstrate a partial and ongoing recovery, indicating that muscle atrophy and denervation are not yet complete due to insufficient nerve regeneration. Additional evaluation at later timepoints would probably reveal higher amplitude and lower latency values. Similar electrophysiological evaluation results were observed previously in this species, with a progressive recovery of amplitude and latency parameter values over the recovery time, but without approaching the values of the UC group, particularly in amplitude (Contreras et al., 2023). Here it was also confirmed that for longer timepoints, there was a continuous recovery of the evaluated parameters.

Stereological evaluation performed 24 weeks after injury induction allowed to identify different therapeutic performances regarding the ability to promote regeneration and ultrastructural reorganization of injured common peroneal nerves. Although signs of nerve regeneration were observed in all groups, outcomes varied according to the therapeutic approach considered, and as previously indicated, the inability to analyze some of the nerves harvested limited the establishment of more profound conclusions. All therapeutic groups displayed signs of microfasciculation, characterized by smaller fibers with thinner myelin sheaths compared to uninjured controls. This phenomenon likely reflects a transitional phase of nerve regeneration, where smaller, immature fibers precede the development of larger, fully functional axons, and indicates the effective occurrence of nerve regeneration. The EtE group consistently presented the best results in the parameters number of fibers, axonal diameter, fiber diameter and myelin thickness, confirming once again that physical juxtaposition between the nerve ends can promote earlier axonal reconnection, which is only achieved later with the application of NGCs where an interneuronal gap is created. Among all these parameters, nerve fiber diameter is one of the most important as it relates myelin sheath thickness to axonal diameter and is a determining factor in nerve conduction velocity (Ikeda and Oka, 2012). Interestingly, this superior performance of the EtE group was not recorded in previous studies in the rat model using similar therapeutic approaches (Alvites R. D. et al., 2022). In the parameter fiber density, the NGC group, which is the one with the worst results in the remaining parameters, presents the highest value. This result indicates that this group is in an earlier stage of the regenerative process, in which a high number of regenerated axons derived from the growth of axonal sprouts between the two nerve ends can still be observed, and in which a selection of definitive nerve fibers with abrasion of the dysfunctional ones has not yet occurred. The g-ratio is an axonal geometric constant that establishes a relationship between the axonal cross-sectional size and the level of myelination, since by calculating the ratio between the inner axonal diameter and the total diameter of the nerve fiber (*d*/*D*), a determination of the quality of axonal myelination is obtained. Together with the axonal diameter, the g-ratio is thus an indicator of the speed of neural conduction (Bouhrara et al., 2021). It is established that the optimal g-ratio values should be located between 0.6 and 0.75 (Berman et al., 2018), and small variations in the thickness of the myelin sheaths will be sufficient for g-ratio values to be above or below the ideal range, translating into variations in conduction velocity and consequent functional disturbances. In this case, in all therapeutic groups the g-ratio value is within the ideal reference range, with no statistical differences observed with the UC. The highest cross-section area was also observed in the EtE group. The cross-section area does not necessarily translate into a better regenerative process, since it can often be associated with phenomena of residual fibrosis, adipose infiltration or peripheral edema, which may instead translate into functional impairments. In any case, no statistical differences were observed with the UC group for this parameter. The outcomes resulting from the stereological evaluation of the common peroneal nerves that underwent surgery revealed that all therapeutic options promoted the expected occurrence of nerve regeneration, axonal myelination and neuronal conduction velocity identical to those observed in healthy nerves, which may explain the results recorded in the functional and kinematic evaluations. Although in general the EtE group had a better performance than the groups that received NGC, the reduced number of nerves analyzed, the lack of statistical differences between the therapeutic groups and the consistently lower values than those observed in the UC group do not allow to establish any therapeutic option as unequivocally more beneficial. The application of NGC seems to lead to a worse ultrastructural reorganization than the combination with CM, revealing advantages in the therapeutic application of the paracrine factors. In previous studies in the rat model, the application of biomaterials and their combination with CM led to a better performance than the application of the EtE suture (Alvites R. D. et al., 2022). The constraints related to the inability to analyze some of the collected nerves may be related to inadequate fixation after harvesting, not only due to the relatively large diameter of these nerves at 24 weeks after surgery but also due to the large amount of adipose tissue that is macroscopically observed during harvesting (and whose presence is also indicated by ultrasound observations). This adipose tissue, which is observed in healthy tissues and can be aggravated by infiltration associated with surgical manipulation and the regenerative process, acts as a barrier that slows and hinders the penetration of water-based fixatives such as glutaraldehyde, preventing correct manipulation and subsequent analysis of the nerves. New efforts should be made in the future to improve the protocol for harvesting and fixation of ovine nerves, namely, by performing careful dissection and removal of the adipose tissue around the nerve and increasing the fixation time, thus ensuring good nerve fixation and the possibility of maximizing the amount of information obtained *post-mortem*.

One of the most limiting direct consequences after peripheral nerve injury is neurogenic atrophy of the respective effector muscles, which results in changes in motor performance and eventual neuropathic pain (Mehrotra et al., 2024). Surprisingly, in comparison with the nerve stereological evaluation, the NGC group presented the highest values for fiber area and minimum Feret´s diameter, with the EtE group having the worst performance. The values recorded in the NGC group are high enough to allow differences between this group and the others in both parameters, and to prevent differences with the UC group in the minimum Feret´s diameter. These results corroborate the dimensions observed in the muscles of this group in the ultrasound evaluation but appear to contradict the results observed in the nerve stereological evaluation. It is possible that the application of NGCs provides slower axonal regeneration, but with better alignment and structural support, which may lead to a more positive impact on the functional reactivation of the muscles over time. The differences also reinforce the need for the determination of the pro-regenerative capacity of a given treatment to be evaluated using a multimodal approach instead of using a single type of assessment. Once again, the capacity of all therapeutic groups to promote muscle recovery falls short of the values observed in the control group, in a performance that follows similar trends when applying the same treatment in the rat model (Alvites R. D. et al., 2022).

The overall results of this study confirm the pro-regenerative potential of using biomaterials in combination with MSC secretion products in the ovine model, in a scale-up approach after observing promising results in the rat model. Although all therapeutic groups demonstrated varying levels of efficacy in functional and ultrastructural recovery, the presence of evident differences in the recovery patterns is undeniable. Contrary to what was observed in studies carried out in rodents (Alvites R. D. et al., 2022), in this case the application of NGCs rarely unequivocally surpassed the performance achieved by the gold-standard EtE approach, and even comparing the two groups that received NGCs, the addition of CM did not always translate into better results, indicating that the benefits of this therapeutic combination may be context-dependent. The use of guide conduits alone, however, brings benefits to the regenerative process by creating a physical guidance that facilitates axonal alignment and protects the regeneration site from excessive inflammation and scarring, as has been confirmed in previous studies (Mankavi et al., 2023; Cao et al., 2024). Overall, the results of the therapeutic approaches considered are consistently like each other and often fall below the values and results observed in the control group. These more dubious and irregular results than those observed in similar conditions for the rat model confirm the need to test and explore the therapeutic options developed in less complex animal models in larger models such as sheep, whose regenerative complexity is not only closer to that observed in humans but also creates additional challenges and difficulties. The variability in responses underscores the complexity of peripheral nerve repair in these intricate cases and highlights the need for multimodal assessments that integrate functional, structural, and electrophysiological data to provide a comprehensive understanding of therapeutic efficacy. It is necessary to continue exploring and improving new therapeutic approaches for the resolution and promotion of peripheral nerve regeneration after injury.

To enhance the therapeutic potential of the secretome, innovative strategies such as priming and personalized pre-application protocols (Teli et al., 2023) could represent promising advancements to be explored in future works. In the future, CM components, such as exosomes and microvesicles, may also be isolated and studied independently and applied in a highly specific manner, skipping the use of the complete CM and using only its intended components, making therapy even more personalized. Surgically, the use of gaps of greater length will also be important to validate the therapeutic effects of the treatment in more complex scenarios that are more difficult to resolve. Likewise, the limitations and difficulties associated with the application of the CM inside the lumen in the NGC, with the risk of loss and extravasation into neighboring tissues, may in the future be overcome through the simultaneous application of hydrogel prefilling the conduit, as previously performed by the group (Alvites et al., 2021), or the use of a slow particle release system.

## 5 Conclusion

The exploration of biomaterials and cell secretion products to promote peripheral nerve regeneration has been a constant in the field of regenerative medicine. This study, which aimed to compare the pro-regenerative efficacy of the combined use of commercially available neural guide conduits with CM produced from OM-MSCs, emerged following extensive studies carried out in the rat model in which this therapeutic combination proved to be particularly promising.

The results obtained in the *in vivo* and *post-mortem* evaluation after induction of neurotmesis injuries in the common peroneal nerves of the ovine model reveal that all therapeutic options could promote nerve and muscle regeneration effective enough to lead to functional recovery and evident histological and ultrastructural reorganization. However, contrary to what was previously observed in the rat model, the use of biomaterials and their combination with CM did not always surpass the performance of traditional EtE techniques, and none of the therapeutic approaches were able to match the values recorded in healthy animals and tissues.

In conclusion, the results of this study highlight the significant potential of using the OM-MSCs cell CM in combination with biomaterials to promote peripheral nerve regeneration in complex animal models. These findings open new possibilities for therapeutic innovation in regenerative medicine. However, the irregularities observed in the functional and histomorphometric evaluations underscore the multifaceted complexity of nerve regeneration. These inconsistencies not only reflect the challenges inherent to the nerve regeneration process but also emphasize the necessity for further research. Future studies should aim to address these uncertainties, refine the therapeutic protocols, and explore novel approaches to optimize the use of MSCs and their secretion products in promoting more consistent and effective nerve repair and regeneration. The inclusion of an additional control group that would receive the application of basal culture medium (e.g., unconditioned DMEM/F12 or FBS) could be important to determine whether the observed therapeutic effects are exclusively attributable to the bioactive factors secreted by MSCs, eliminating the possible influence of residual components of the culture medium. Longer follow-up times, beyond 24 weeks, may also be effective in revealing more consolidated functional improvements, allowing the identification of late-stage regenerative events and potentially amplifying the differences between therapeutic groups.

## Data Availability

The original contributions presented in the study are included in the article/Supplementary Material, further inquiries can be directed to the corresponding author.

## Abbreviations

CM, Conditioned Medium; CMAP, Compound Muscle Action Potential; EGM, Electromyography; EtE, End-to-end suture; MSCs, Mesenchymal Stem Cells; NGC, Nerve Guidance Conduit; NGC-CM, Nerve Guidance Conduit and Conditioned Medium; OM, Olfactory Mucosa; OM-MSCs, Olfactory Mucosa Mesenchymal Stem Cells; UC, Uninjured control.
