# Supplementary material for: Combined use of olfactory mucosal mesenchymal stem cells conditioned medium and neural guide conduits promotes nerve regeneration in an ovine model
Source: Front Cell Dev Biol. 2025 May 2;13:1598736. doi: 10.3389/fcell.2025.1598736 (PMC12081432; doi:10.3389/fcell.2025.1598736)
Supplement: Supplementary file 1 [file DataSheet1.pdf]

Supplementary Material

Table S1: Values of postural evaluation performed in stationary position. These tests were performed preoperatively (T0), 1 and 2 weeks after neurotmesis (T1 and T2) and from there every two weeks until week 24 (T24). Results are presented as mean and SD. (*n* = number of animals per group).

Postural Evaluation - Stationary Position		Time													
		T0	T1	T2	T4	T6	T8	T10	T12	T14	T16	T18	T20	T22	T24
Group 1: Uninjured Control (*n* = 27)	Mean	1.00	1.00	1.00	1.00	1.00	1.00	1.00	1.00	1.00	1.00	1.00	1.00	1.00	1.00
	SD	0.00	0.00	0.00	0.00	0.00	0.00	0.00	0.00	0.00	0.00	0.00	0.00	0.00	0.00
Group 2: Neurotmesis + End to End (*n* = 6)	Mean	1.00	5.00	5.00	5.00	4.50	4.33	4.00	2.50	1.50	1.00	1.00	1.00	1.00	1.00
	SD	0.00	0.00	0.00	0.00	0.55	0.82	1.10	0.55	0.55	0.00	0.00	0.00	0.00	0.00
Group 3: Neurotmesis + Nerve Guidance Conduit (*n* = 6)	Mean	1.00	5.00	5.00	4.83	4.17	4.17	3.83	3.67	3.50	2.50	2.17	1.67	1.67	1.67
	SD	0.00	0.00	0.00	0.41	0.41	0.75	0.98	1.03	1.22	1.64	1.47	1.03	1.03	1.03
Group 4: Neurotmesis + Nerve Guidance Conduit + Secretome (*n* = 4)	Mean	1.00	5.00	5.00	5.00	4.00	4.00	4.00	3.00	2.00	1.75	1.75	1.50	1.25	1.25
	SD	0.00	0.00	0.00	0.00	0.00	0.00	0.00	0.00	0.00	0.96	0.96	1.00	0.50	0.50

Table S2: Statistical differences observed in postural evaluation in stationary position at 24 weeks. UC: Uninjured Control; EtE: end-to-end suture; NGC: application of Reaxon® NGC; NGC-CM: application of Reaxon® NGC and OM-MSCs CM. * corresponds to 0.01 ≤ *p* < 0.05, ** to 0.001 ≤ *p* < 0.01, *** to 0.0001 ≤ *p* < 0.001, and **** to *p* < 0.0001, ns = no statistically significant differences.

Postural Evaluation – Stationary Position				
	UC	EtE	NGC	NGC-CM
UC		ns	ns	ns
EtE			ns	ns
NGC				ns
NGC-CM				

Table S3: Values of proprioceptive assessment: static repositioning. These tests were performed preoperatively (T0), 1 and 2 weeks after neurotmesis (T1 and T2) and from there every two weeks until week 24 (T24). Results are presented as mean and SD. (*n* = number of animals per group).

Proprioception: Static Repositioning		Time													
		T0	T1	T2	T4	T6	T8	T10	T12	T14	T16	T18	T20	T22	T24
Group 1: Uninjured Control (*n* = 27)	Mean	1.00	1.00	1.00	1.00	1.00	1.00	1.00	1.00	1.00	1.00	1.00	1.00	1.00	1.00
	SD	0.00	0.00	0.00	0.00	0.00	0.00	0.00	0.00	0.00	0.00	0.00	0.00	0.00	0.00
Group 2: Neurotmesis + End to End (*n* = 6)	Mean	1.00	6.00	5.00	4.33	3.67	3.67	3.33	2.83	3.00	2.50	2.00	2.33	1.67	1.33
	SD	0.00	0.00	0.00	1.63	1.37	0.82	1.21	0.41	0.00	0.55	0.00	0.82	0.52	0.52
Group 3: Neurotmesis + Nerve Guidance Conduit (*n* = 6)	Mean	1.00	5.33	5.17	4.67	4.50	3.83	3.17	3.17	2.33	2.33	2.17	2.33	1.67	1.67
	SD	0.00	0.52	0.75	1.03	1.22	1.83	1.47	1.47	2.07	1.75	1.47	1.37	1.03	1.03
Group 4: Neurotmesis + Nerve Guidance Conduit + Secretome (*n* = 4)	Mean	1.00	5.00	4.25	4.00	3.50	2.50	3.00	2.25	1.50	1.25	1.25	1.25	1.25	1.00
	SD	0.00	0.00	0.50	0.00	0.58	0.58	0.00	0.50	1,00	0.50	0.50	0.50	0.50	0.00

Table S4: Statistical differences observed in proprioceptive assessment: static repositioning at 24 weeks. UC: Uninjured Control; EtE: end-to-end suture; NGC – application of Reaxon® NGC; NGC-CM: application of Reaxon® NGC and OM-MSCs CM. * corresponds to $0.01 \leq p < 0.05$, ** to $0.001 \leq p < 0.01$, *** to $0.0001 \leq p < 0.001$, and **** to $p < 0.0001$, ns = no statistically significant differences.

Proprioceptive Assessment: Static Repositioning				
	UC	EtE	NGC	NGC-CM
UC		ns	ns	ns
EtE			ns	ns
NGC				ns
NGC-CM				

Table S5: Values of proprioceptive assessment: dynamic repositioning. These tests were performed preoperatively (T0), 1 and 2 weeks after neurotmesis (T1 and T2) and from there every two weeks until week 24 (T24). Results are presented as mean and SD. (n = number of animals per group).

Proprioception: Dynamic repositioning		Time													
		T0	T1	T2	T4	T6	T8	T10	T12	T14	T16	T18	T20	T22	T24
Group 1: Uninjured Control (n = 27)	Mean	1.00	1.00	1.00	1.00	1.00	1.00	1.00	1.00	1.00	1.00	1.00	1.00	1.00	1.00
	SD	0.00	0.00	0.00	0.00	0.00	0.00	0.00	0.00	0.00	0.00	0.00	0.00	0.00	0.00
Group 2: Neurotmesis + End to End (n = 6)	Mean	1.00	6.00	5.50	5.50	4.83	4.50	3.67	3.50	3.00	2.50	2.33	2.17	1.67	1.67
	SD	0.00	0.00	0.55	0.55	0.41	0.84	0.52	0.84	0.90	0.84	0.52	0.75	0.52	0.52
Group 3: Neurotmesis + Nerve Guidance Conduit (n = 6)	Mean	1.00	5.33	5.33	4.33	4.00	4.00	3.17	3.33	2.50	2.67	2.33	2.67	2.00	1.67
	SD	0.00	0.52	0.52	1.37	1.55	0.90	1.47	1.51	1.97	1.97	2.07	1.03	1.55	1.03
Group 4: Neurotmesis + Nerve Guidance Conduit + Secretome (n = 4)	Mean	1.00	5.00	4.75	4.00	3.50	3.00	3.00	2.25	1.50	1.25	1.25	1.25	1.25	1.00
	SD	0.00	0.00	0.50	0.00	0.58	0.00	0.00	0.50	1.00	0.50	0.50	0.50	0.50	0.00

Table S6: Statistical differences observed in proprioceptive assessment: dynamic repositioning at 24 weeks. UC: Uninjured Control; EtE: end-to-end suture; NGC: application of Reaxon® NGC; NGC-CM: application of Reaxon® NGC and OM-MSCs CM. * corresponds to $0.01 \leq p < 0.05$, ** to $0.001 \leq p < 0.01$, *** to $0.0001 \leq p < 0.001$, and **** to $p < 0.0001$, ns = no statistically significant differences.

Proprioceptive Assessment: Dynamic Repositioning				
	UC	EtE	NGC	NGC-CM
UC		ns	ns	ns
EtE			ns	ns
NGC				ns
NGC-CM				

Table S7: Values of spinal reflexes assessed using the withdrawal reflex. These tests were performed preoperatively (T0), 1 and 2 weeks after neurotmesis (T1 and T2) and from there every two weeks until week 24 (T24). Results are presented as mean and SD. (*n* = number of animals per group).

Withdrawal reflex		Time													
		T0	T1	T2	T4	T6	T8	T10	T12	T14	T16	T18	T20	T22	T24
Group 1: Uninjured Control (*n* = 27)	Mean	3.00	3.00	3.00	3.00	3.00	3.00	3.00	3.00	3.00	3.00	3.00	3.00	3.00	3.00
	SD	0.00	0.00	0.00	0.00	0.00	0.00	0.00	0.00	0.00	0.00	0.00	0.00	0.00	0.00
Group 2: Neurotmesis + End to End (*n* = 6)	Mean	3.00	1.00	1.00	1.00	1.33	1.50	1.67	2.17	2.17	2.67	2.67	2.67	2.67	2.67
	SD	0.00	0.00	0.00	0.00	0.52	0.55	0.52	0.75	0.99	0.52	0.52	0.52	0.52	0.52
Group 3: Neurotmesis + Nerve Guidance Conduit (*n* = 6)	Mean	3.00	1.00	1.00	1.00	1.17	1.67	2.00	2.00	1.67	2.17	2.33	2.50	2.50	2.50
	SD	0.00	0.00	0.00	0.00	0.41	0.52	0.00	0.00	0.52	0.41	0.52	0.55	0.55	0.55
Group 4: Neurotmesis + Nerve Guidance Conduit + Secretome (*n* = 4)	Mean	3.00	1.00	1.00	1.00	1.00	1.25	1.75	1.75	1.75	2.5	2.5	2.25	2.25	2.75
	SD	0.00	0.00	0.00	0.00	0.00	0.50	0.50	0.50	0.50	0.58	0.58	0.50	0.50	0.50

Table S8: Statistical differences observed in spinal reflexes assessed through the withdrawal reflex at 24 weeks. UC: Uninjured Control; EtE: end-to-end suture; NGC: application of Reaxon® NGC; NGC-CM: application of Reaxon® NGC and OM-MSCs CM. * corresponds to $0.01 \leq p < 0.05$, ** to $0.001 \leq p < 0.01$, *** to $0.0001 \leq p < 0.001$, and **** to $p < 0.0001$, ns = no statistically significant differences.

Withdrawal reflex

	UC	EtE	NGC	NGC-CM
**UC**		**ns**	**ns**	**ns**
**EtE**			**ns**	**ns**
**NGC**				**ns**
**NGC-CM**				

Table S9: Spatiotemporal parameters determined in the different therapeutic groups. Results are presented as mean and SD.

SPATIO-TEMPORAL PARAMETERS - EtE

Speed	0.846 m/s	0.000 Statures/s
Stride	Wid : NO_DATA (0)	Len(52) 0.626±0.088m
Cycle Time	Computed: 0.762 s	Actual (120) 0.740±0.124 s
Measure±StdDev (Count)		Measure±StdDev (Count)
Left : NO_DATA (0)	Step Length	Right : NO_DATA (0)
Left : 0.408±0.049 s (58)	Step Time	Right 0.366±0.054 s (59)
Left : 0.485±0.078 s (61)	Stance Time	Right : 0.466±0.106 s (85)
Left : 0.285±0.036 s (65)	Swing Time	Right : 0.248±0.039 s (91)
Left : 149.369±20.364 (58)	Steps Per Minute	Right : 167.430±26.013 (59)
Left : 0.108±0.039 s (67)	Initial DBL Support	Right : 0.122±0.043 s (66)
Double Limb Support Time (133)		0.229±0.082 s

SPATIO-TEMPORAL PARAMETERS - NGC

Speed	0.805 m/s	0.000 Statures/s
Stride	Wid : NO_DATA (0)	Len(15) 0.719±0.137m
Cycle Time	Computed: 0.903 s	Actual (30) 0.893±0.103 s
Measure±StdDev (Count)		Measure±StdDev (Count)
Left : NO_DATA (0)	Step Length	Right : NO_DATA (0)
Left : 0.488±0.055 s (20)	Step Time	Right 0.407±0.068 s (16)
Left : 0.549±0.110 s (19)	Stance Time	Right : 0.614±0.092 s (21)
Left : 0.333±0.066 s (26)	Swing Time	Right : 0.294±0.034 s (20)
Left : 124.473±13.102 (20)	Steps Per Minute	Right : 151.638±27.426 (16)
Left : 0.128±0.058 s (21)	Initial DBL Support	Right : 0.148±0.099 s (24)
Double Limb Support Time (45)		0.276±0.156 s

SPATIO-TEMPORAL PARAMETERS - NGC-CM

Speed	0.745 m/s	0.000 Statures/s
Stride	Wid(2) 0.238±0.124m	Len(22) 0.642±0.151m
Cycle Time	Computed: 0.866 s	Actual (48) 0.862±0.092 s
Measure±StdDev (Count)		Measure±StdDev (Count)
Left : NO_DATA (0)	Step Length	Right : NO_DATA (0)
Left : 0.464±0.059 s (32)	Step Time	Right 0.403±0.071 s (26)
Left : 0.537±0.084 s (24)	Stance Time	Right : 0.610±0.111 s (33)
Left : 0.323±0.064 s (33)	Swing Time	Right : 0.238±0.036 s (38)
Left : 131.490±17.221 (32)	Steps Per Minute	Right : 153.605±28.196 (26)
Left : 0.166±0.089 s (31)	Initial DBL Support	Right : 0.138±0.048 s (34)
Double Limb Support Time (65)		0.304±0.137 s

Table S10: Values of nerve diameter measured using ultrasound. These measurements were performed preoperatively (T0), and 4 weeks (T4), 12 weeks (T12) and 24 weeks (T24) after surgical intervention. Results are presented as mean and SD. (n = number of animals per group).

**Nerve Diameter**		**T0**	**T4**	**T12**	**T24**
**Group 1: Uninjured Control (*n* = 16)**	Mean	1.36	1.36	1.36	1.36
	SD	0.09	0.09	0.09	0.09
**Group 2: Neurotmesis + End to End (*n* = 4)**	Mean	1.36	2.82	2.93	2.60
	SD	0.09	0.33	0.11	0.46
**Group 3: Neurotmesis + Nerve Guidance Conduit (*n* = 4)**	Mean	1.36	2.52	3.64	3.48
	SD	0.09	0.77	0.59	0.22
**Group 4: Neurotmesis + Nerve Guidance Conduit + Secretome (*n* = 4)**	Mean	1.36	2.82	3.35	3.08
	SD	0.09	0.64	0.54	0.62

Table S11: Statistical differences observed in nerve diameter measured using ultrasound at 24 weeks. UC: Uninjured Control; EtE: end-to-end suture; NGC: application of Reaxon® NGC; NGC-CM: application of Reaxon® NGC and OM-MSCs CM. * corresponds to $0.01 \leq p < 0.05$, ** to $0.001 \leq p < 0.01$, *** to $0.0001 \leq p < 0.001$, and **** to $p < 0.0001$, ns = no statistically significant differences.

Nerve Diameter

	**UC**	**EtE**	**NGC**	**MGC + Secretome**
**UC**		*	***	*
**EtE**			ns	ns
**NGC**				ns
**NGC-CM**				

Table S12: Values of muscle width and thickness measured using ultrasound. These measurements were performed preoperatively (T0), and 4 weeks (T4), 12 weeks (T12) and 24 weeks (T24) after surgical intervention. Results are presented as mean and SD. (n = number of animals per group).

Muscle Width and Thickness			T0	T4	T12	T24
Group 1: Uninjured Control (*n* = 16)	Thickness	Mean	7.60	7.60	7.60	7.60
		SD	0.58	0.58	0.58	0.58
	Width	Mean	18.74	18.74	18.74	18.74
		SD	3.75	3.75	3.75	3.75
Group 2: Neurotmesis + End to End (*n* = 4)	Thickness	Mean	7.60	6.10	6.50	7.25
		SD	0.58	1.35	0.08	0.39
	Width	Mean	18.74	16.3	15.97	17.58
		SD	3.75	1.69	0.53	0.88
Group 3: Neurotmesis + Nerve Guidance Conduit (*n* = 4)	Thickness	Mean	7.60	7.70	7.50	7.60
		SD	0.58	0.33	0.42	0.65
	Width	Mean	18.74	16.93	17.10	18.07
		SD	3.75	1.04	0.00	0.29
Group 4: Neurotmesis + Nerve Guidance Conduit + Secretome (*n* = 4)	Thickness	Mean	7.60	6.80	6.57	6.70
		SD	0.58	0.65	0.34	0.42
	Width	Mean	18.74	17.77	15.93	17.27
		SD	3.75	0.33	4.55	4.05

Table S13: Statistical differences observed in muscle thickness (**a**) and width (**b**) measured using ultrasound at 24 weeks. UC: Uninjured Control; EtE: end-to-end suture; NGC: application of Reaxon® NGC; NGC-CM: application of Reaxon® NGC and OM-MSCs CM. * corresponds to $0.01 \leq p < 0.05$, ** to $0.001 \leq p < 0.01$, *** to $0.0001 \leq p < 0.001$, and **** to $p < 0.0001$, ns = no statistically significant differences.

a) Muscle Thickness

	UC	EtE	CMOM	ECMOM
UC		ns	ns	ns
EtE			ns	ns
NGC				ns
NGC-CM				

b) Muscle Width

	UC	EtE	NGC	MGC + Secretome
UC		ns	ns	ns
EtE			ns	ns
NGC				ns
NGC-CM				

Table S14: Values of amplitude and latency determined through electrophysiological evaluation. These measurements were performed preoperatively (T0), and 4 weeks (T4), 12 weeks (T12) and 24 weeks (T24) after surgical intervention. Results are presented as mean and SD. (n = number of animals per group).

Electrophysiological Evaluation			T0	T4	T12	T24
Group 1: Uninjured Control (*n* = 16)	Amplitude	Mean	22.68	22.68	22.68	22.68
		SD	1.37	1.37	1.37	1.37
	Latency	Mean	3.79	3.79	3.79	3.79
		SD	0.85	0.85	0.85	0.85
Group 2: Neurotmesis + End to End (*n* = 4)	Amplitude	Mean	22.68	3.13	13.20	16.30
		SD	1.37	1.72	1.73	1.30
	Latency	Mean	3.80	10.58	11.59	6.18
		SD	0.85	4.95	6.48	2.06
Group 3: Neurotmesis + Nerve Guidance Conduit (*n* = 4)	Amplitude	Mean	22.68	0.00	4.63	14.48
		SD	1.37	0.00	2.44	2.60
	Latency	Mean	3.79	nd	15.55	10.56
		SD	0.85	nd	2.67	4.96
Group 4: Neurotmesis + Nerve Guidance Conduit + Secretome (*n* = 4)	Amplitude	Mean	22.68	0.00	5.85	17.13
		SD	1.37	0.00	3.18	1.36
	Latency	Mean	3.79	nd	16.18	5.43
		SD	0.85	nd	2.06	1.36

Table S15: Statistical differences observed in amplitude (**a**) and latency (**b**) determined through electrophysiological evaluation at 24 weeks. UC: Uninjured Control; EtE: end-to-end suture; NGC: application of Reaxon® NGC; NGC-CM: application of Reaxon® NGC and OM-MSCs CM. * corresponds to $0.01 \leq p < 0.05$, ** to $0.001 \leq p < 0.01$, *** to $0.0001 \leq p < 0.001$, and **** to $p < 0.0001$, ns = no statistically significant differences.

a) Amplitude

	UC	EtE	NGC	MGC + Secretome
UC		**	*	**
EtE			ns	ns
NGC				ns
NGC-CM				

b) Latency

	UC	EtE	CMOM	ECMOM
UC		ns	ns	ns
EtE			ns	ns
NGC				ns
NGC-CM				

Table S16: Stereological quantitative assessment. The different parameters considered were evaluated in the regenerated common peroneal nerve at week 24 (T24) after neurotmesis. Results are presented as mean and SD (n = number of animals per group).

Stereological Quantitative Assessment		Density	Total number	Axon diameter (d)	Fiber diameter (D)	Myelin thickness (M)	M/d	D/d	d/D (g-ratio)	Cross-sectional area (mm^2)
Group 1: Uninjured Control	Mean	9245	17401	5.58	9.28	1.85	0.33	0.06	0.61	1.94
(*n* = 5)	SD	1387	3560	0.69	1.25	0.36	1.67	0.11	0.05	0.6
Group 2: Neurotmesis + End to End	Mean	10041	21368	3.43	5.16	0.86	0.25	0.02	0.65	2.09
(*n* = 3)	SD	1585	7208	0.12	0.02	0.05	1.5	0.05	0.03	0.39
Group 3: Neurotmesis + Nerve Guidance Conduit	Mean	13933	15848	2.5	3.75	0.63	0.25	0.01	0.65	1.12
(*n* = 2)	SD	1239	5441	0.36	0.48	0.06	1.5	0.02	0.00	0.29
Group 4: Neurotmesis + Nerve Guidance Conduit + Secretome	Mean	10408	17968	2.66	4.15	0.74	0.28	1.56	0.62	1.72
(*n* = 2)	SD	594	2497	0.85	1.05	0.1	0.12	1.24	0.05	0.14

Table S17: Statistical differences in the nerve stereological analysis: **a)** Density of fibers; **b)** Total number of fibers; **c)** Axon diameter; **d)** Fiber diameter; **e)** Myelin thickness; **f)** g-ratio; **g)** Cross sectional area. (ns = no statistically significant differences).

a) **Density**

	UC	EtE	NGC	NGC-CM
UC		ns	ns	ns
EtE			ns	ns
NGC				ns
NGC-CM				

b) **Total number**

	UC	EtE	NGC	NGC-CM
UC		ns	ns	ns
EtE			ns	ns
NGC				ns
NGC-CM				

c) **Axon diameter (d)**

	UC	EtE	NGC	NGC-CM
UC		ns	*	*
EtE			ns	ns
NGC				ns
NGC-CM				

d) **Fiber diameter (D)**

	UC	EtE	NGC	NGC-CM
UC		ns	*	ns
EtE			ns	ns
NGC				ns
NGC-CM				

e) **Myelin thickness (M)**

	UC	EtE	NGC	NGC-CM
UC		ns	*	ns
EtE			ns	ns
NGC				ns
NGC-CM				

f) **d/D (g-ratio)**

	UC	EtE	NGC	NGC-CM
UC		ns	ns	ns
EtE			ns	ns
NGC				ns
NGC-CM				

g) **Cross-sectional area (mm^2)**

	UC	EtE	NGC	NGC-CM
UC		ns	ns	ns
EtE			ns	ns
NGC				ns
NGC-CM				

Table S18: Muscle histomorphometric analysis. The different parameters considered were evaluated in the cranial tibial muscle at week 24 (T24) after neurotmesis. Results are presented as mean and SD (n = number of animals per group).

Muscle Histomorphometric ssessment		Fiber Area (μm^2)	Minimum Feret's Diameter (mm)
Group 1: Uninjured Control	Mean	1455055	37312
(*n* = 15)	SD	689830	35563
Group 2: Neurotmesis + End to End	Mean	1033577	31251
(*n* = 4)	SD	517882	8077
Group 3: Neurotmesis + Nerve Guidance Conduit	Mean	1278557	34834
(*n* = 4)	SD	634815	35452
Group 4: Neurotmesis + Nerve Guidance Conduit + Secretome	Mean	1038731	30381
(*n* = 4)	SD	719978	10902

Table S19: Statistical differences in the muscle histomorphometric analysis : **a)** Fiber Area **b)** Minimum Feret's Diameter (ns = no statistically significant differences).

a) **Fiber Area**

	UC	EtE	NGC	NGC-CM
UC		****	****	****
EtE			****	ns
NGC				****
NGC-CM				

b) **Minimum Feret's Diameter**

	UC	EtE	NGC	NGC-CM
UC		****	**	****
EtE			**	ns
NGC				*
NGC-CM				

Table S20: Muscle Mass Lost in the cranial tibial muscles of the different therapeutic groups compared to healthy contralateral muscles. Healthy Control and Lesion values are expressed in grams (g).

Lost of Muscle Mass		Healthy Control	Lesion	Ratio	% of loss
Group 2: Neurotmesis + End to End	Mean	61.16	57.56	83.18	16.82
(*n* = 4)	SD	5.26	4.45	10.33	
Group 3: Neurotmesis + Nerve Guidance Conduit	Mean	64.98	63.37	93.05	6.95
(*n* = 4)	SD	2.49	3.60	7.45	
Group 4: Neurotmesis + Nerve Guidance Conduit + Secretome	Mean	66.54	60.51	78.16	21.84
(*n* = 4)	SD	3.30	0.92	10.49	
